# Rab11-FIP1C and Rab14 Direct Plasma Membrane Sorting and Particle Incorporation of the HIV-1 Envelope Glycoprotein Complex

**DOI:** 10.1371/journal.ppat.1003278

**Published:** 2013-04-04

**Authors:** Mingli Qi, Janice A. Williams, Hin Chu, Xuemin Chen, Jaang-Jiun Wang, Lingmei Ding, Ehiole Akhirome, Xiaoyun Wen, Lynne A. Lapierre, James R. Goldenring, Paul Spearman

**Affiliations:** 1 Department of Pediatrics, Children's Healthcare of Atlanta and Emory University School of Medicine, Atlanta, Georgia, United States of America; 2 Departments of Surgery and Cell and Developmental Biology, Epithelial Biology Center, Vanderbilt University School of Medicine, Nashville, Tennessee, United States of America; University of North Carolina at Chapel Hill, United States of America

## Abstract

The incorporation of the envelope glycoprotein complex (Env) onto the developing particle is a crucial step in the HIV-1 lifecycle. The long cytoplasmic tail (CT) of Env is required for the incorporation of Env onto HIV particles in T cells and macrophages. Here we identify the Rab11a-FIP1C/RCP protein as an essential cofactor for HIV-1 Env incorporation onto particles in relevant human cells. Depletion of FIP1C reduced Env incorporation in a cytoplasmic tail-dependent manner, and was rescued by replenishment of FIP1C. FIP1C was redistributed out of the endosomal recycling complex to the plasma membrane by wild type Env protein but not by CT-truncated Env. Rab14 was required for HIV-1 Env incorporation, and FIP1C mutants incapable of binding Rab14 failed to rescue Env incorporation. Expression of FIP1C and Rab14 led to an enhancement of Env incorporation, indicating that these trafficking factors are normally limiting for CT-dependent Env incorporation onto particles. These findings support a model for HIV-1 Env incorporation in which specific targeting to the particle assembly microdomain on the plasma membrane is mediated by FIP1C and Rab14.

## Author Summary

Enveloped viruses must develop strategies to ensure that a sufficient quantity of their receptor-binding envelope proteins are incorporated onto the surface of viruses as they form. The HIV envelope glycoprotein is specifically incorporated onto assembling virions in relevant cells such as T lymphocytes in a manner that requires its long cytoplasmic tail. The mechanism underlying this specific incorporation has remained unknown. Here, we identify a cellular trafficking pathway that is required for the incorporation of HIV envelope onto virions. A combination of the adaptor protein Rab11-FIP1C and Rab14 directs the envelope protein onto assembling virions, and loss of either of these host factors results in the production of virus particles lacking envelope. We also found that FIP1C was required for replication in T cell lines. This study identifies a trafficking complex required for HIV envelope incorporation and for the formation of infectious HIV particles.

## Introduction

HIV-1 particles assemble on the plasma membrane of infected human cells. The underlying shell of the developing viral particle is formed by the Pr55^Gag^ polyprotein (Gag), which is translated deep within the cytoplasm of cells and reaches the plasma membrane by an unknown mechanism [1], [2], [3]. Gag binds to the viral genomic RNA through its nucleocapsid (NC) region and is responsible for packaging of RNA into the developing particle. A -1 ribosomal frameshift results in the formation of the Gag-Pol precursor protein in a molar ratio of 20∶1 Gag to Gag-Pol [4]. Gag, Gag-Pol, and the associated viral genomic RNA traffic together to the particle assembly site on the plasma membrane. The envelope glycoprotein complex (Env) of HIV-1 is simultaneously incorporated onto the lipid membrane of the developing particle, and yet must travel via a very different route to reach the assembly site.

Env is synthesized on ribosomes associated with the endoplasmic reticulum as a precursor protein, gp160 [5]. Trimerization of gp160 is required for exit from the ER [6]. During transit through the Golgi, gp160 becomes heavily glycosylated and the precursor subunits are cleaved by furin-like proteases to create gp41 (TM) and gp120 (SU) subunits. A trimer of heterodimers of TM and SU forms the functional Env glycoprotein complex. Once the mature complex reaches the cell surface, it is either incorporated onto budding virions or rapidly internalized [7], [8], [9], [10]. In contrast to the dense concentration of envelope glycoprotein spikes on the surface of orthomyxoviruses [11], paramyxoviruses [12], herpesviruses [13], and the relative abundance of envelope spikes on gammaretroviruses [14], lentiviral particles incorporate a very limited number of envelope proteins. Estimates from electron tomography studies reveal an average of 8–14 trimers per virion particle when virions with full-length HIV envelopes are examined [15], [16]. SIV strains with truncations in the cytoplasmic tail (CT) have been noted to incorporate 70–79 trimers and have been widely employed in cryoEM studies of the envelope spike [15], [16], [17]. The reasons for the paucity of Env trimers on lentiviral particles in the absence of mutant CT domains remain unknown.

HIV and SIV Env proteins incorporate long CTs, averaging about 150 amino acids in length. The Env CT has been the subject of numerous studies, yet a complete understanding of how the CT mediates incorporation onto budding virions remains elusive. The Env CT includes a membrane-proximal YXXφ motif that mediates clathrin- and AP2-dependent endocytosis [8], [9], [18], [19], [20] and a number of additional tyrosine-based and dileucine motifs that have been implicated in endocytosis and trafficking of Env [21]. A YW^802^ motif was reported to interact with TIP47, and by this interaction mediate Env interaction with Gag and incorporation onto particles [22]. Murakami and Freed reported that the long CT of Env is required for particle incorporation into virions and for viral replication in T cell lines and primary cells [23]. This report established the cell type-specific, tail-dependent incorporation onto virions, suggesting that host factors may mediate Env incorporation and be differentially expressed in distinct cell types.

The Rab11a Family Interacting Proteins (FIPs) are effector molecules that bind Rab11 family members and mediate sorting of cargo from the endosomal recycling compartment to the plasma membrane [24], [25]. We evaluated Rab11-FIPs as potential mediators of HIV-1 Env incorporation into particles. We identified Rab11-FIP1C/RCP as a specific host cellular factor mediating the delivery of HIV-1 Env onto virion particles in a CT-dependent manner. FIP1C-dependent Env incorporation was not mediated through interaction with Rab11a, but instead required interaction with Rab14.

## Results

### The limitation to virion Env incorporation is cytoplasmic tail-dependent

**Figure 1:**
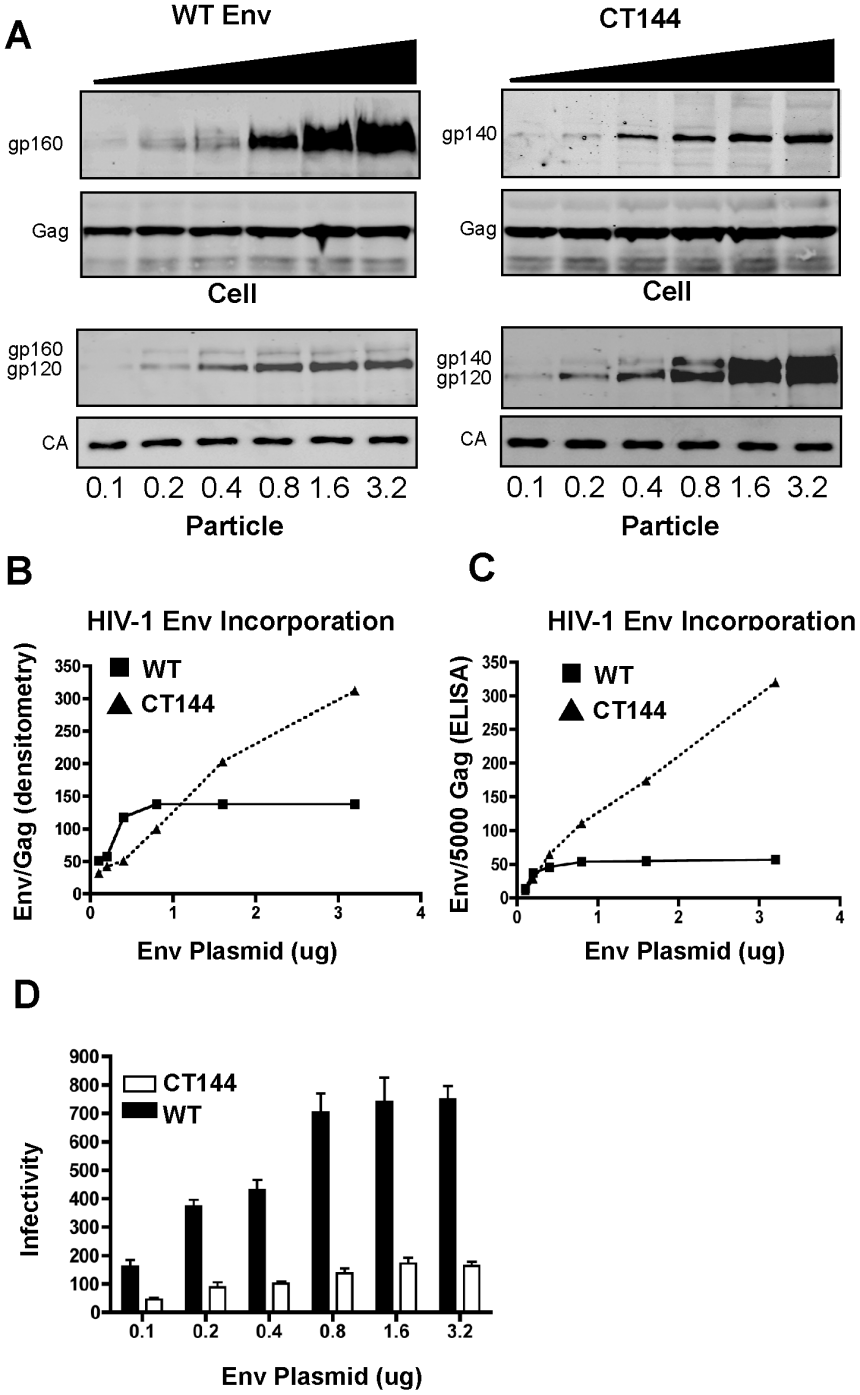
The incorporation of Env onto HIV-1 particles is saturable in a cytoplasmic tail-dependent fashion. (A) Increasing amount of wildtype and the tailless mutant (CT144) of NL4-3 Env protein expressing plasmids were co-transfected with fixed amount of pNL4.3Env- proviral plasmid into HeLa cells. Progeny viral particles were harvested two days after transfection through a 20% sucrose cushion and the particles were lysed with SDS loading buffer. Proteins were analyzed by immunoblot using Env and CA specific antibodies. (B) The intensity of each Env band and Gag band in the particle immunoblot shown in A was quantified by Licor software. Background-subtracted pixel intensity values were plotted on the graph as Env band intensity versus CA band intensity. (C) The absolute quantity of Env and CA/P24 on particles was measured by gp120 ELISA and p24 ELISA. The number of Env molecules per particle was calculated as an approximation, assuming that 5000 CA molecules are present in one HIV-1 virion. (D) The infectivity of the progeny viral particles was measured using TZM-bl reporter cells. Infectivity is plotted as number of blue cells per nanogram of p24 antigen used in the infection assay.

The limited number of Env trimers on HIV-1 particles suggested to us that there is an inherent limitation to Env incorporation that is unrelated to the production of Env protein in the cell. To directly test this hypothesis, we expressed a constant amount of Gag expression plasmid together with an increasing amount of full-length or cytoplasmic tail-truncated Env. We used the CTdel-144 Env (CT144) [23], which retains only 6 cytoplasmic residues of the tail, in order to determine if the cytoplasmic tail was important in limiting the amount of Env on the particle. Expression of full-length Env in cells increased sequentially with increasing amount of plasmid transfected. The amount of Env on released HIV-1 particles did not continue to increase, however, but instead reached a plateau at levels of 0.8 µg or more of transfected DNA (Fig. 1A, WT Env). CT144 expression in cells increased similarly, but incorporation onto HIV-1 particles continued in a linear fashion without apparent saturation (Fig. 1A, CT144). The saturation of full-length Env incorporation was further documented by quantitation of Env/Gag ratio in released particles as measured by densitometry (Fig. 1B) or by gp120 and p24 ELISA (Fig. 1C). We confirmed that the pelletable Env observed in these experiments was particle-associated and not due to overexpression of Env and release on microvesicles (Fig. S1). Despite the ability to incorporate Env at higher levels upon overexpression, particles bearing CT144 Env were consistently less infectious as measured using TZM-bl indicator cells (Fig. 1D). A plateau in particle infectivity conferred by WT Env was noted at the same level of Env expression found to be saturating by Western blot or ELISA analysis (Fig. 1D). These results indicated to us that the cytoplasmic tail of Env confers a limitation to Env trimer incorporation onto HIV-1 particles.

### Rab11-FIP1C is required for Env incorporation onto virions

**Figure 2:**
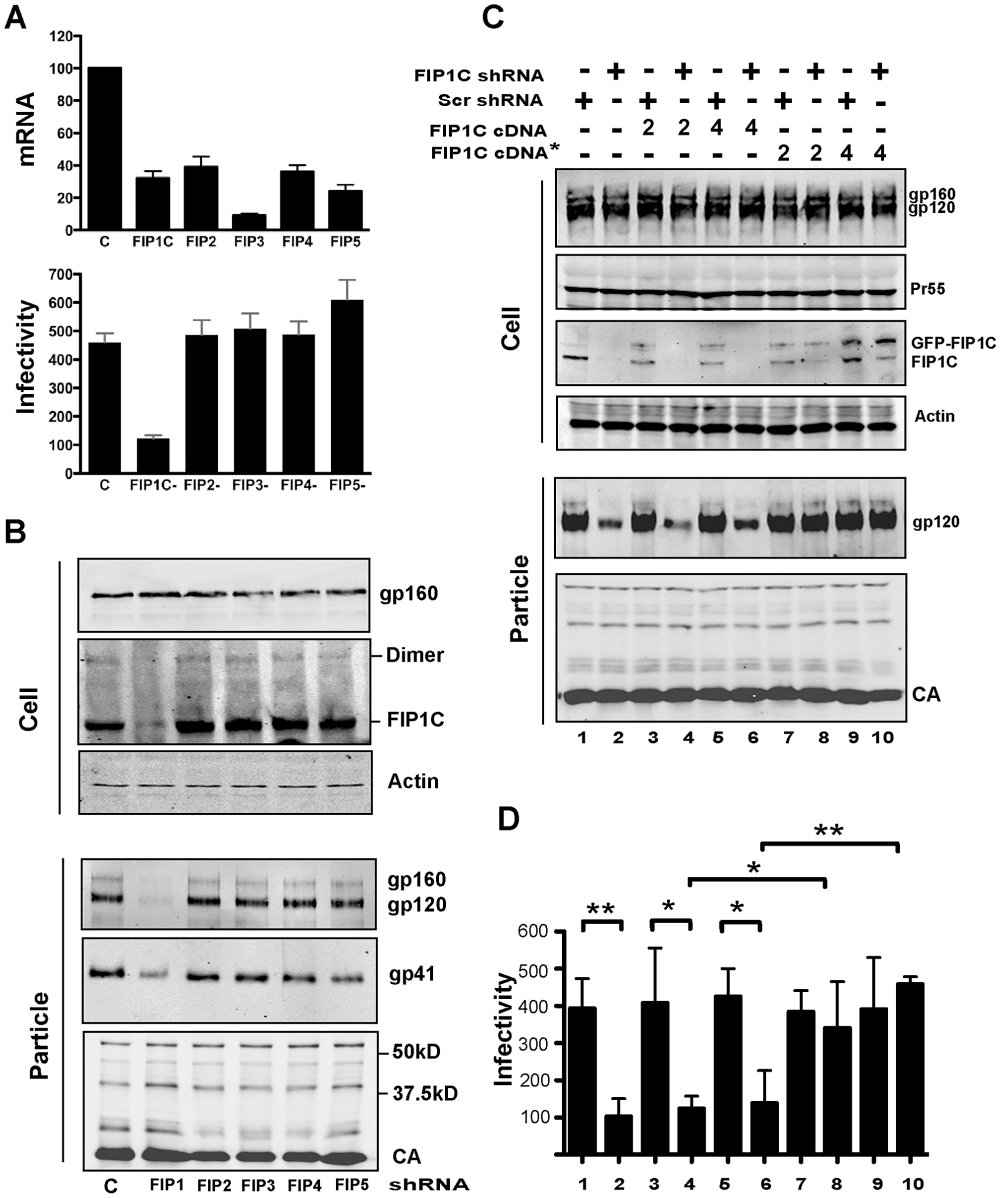
FIP1C depletion and HIV-1 Env incorporation in HeLa cells. (A) The efficiency of depletion of Rab11a-FIPs was measured by real time PCR (top). Infectivity of released particles following depletion of each FIP family member is shown below. (B) Depletion of FIP1C was confirmed at the protein level by immunoblotting with a FIP1C-specific antiserum. Env levels in both cell lysates and virions harvested from the FIP-depleted HeLa cells were assayed by immunoblotting. (C) Restoration of Env incorporation following shRNA-mediated depletion. Two constructs were used in this experiment: FIP1C cDNA is an shRNA sensitive GFP-tagged plasmid; while FIP1C* includes silent mutations in the shRNA target sequence rendering it shRNA-resistant. 2 ug and 4 ug of each FIP1C construct were used in the repletion assay as indicated at the top of the blot. (D) Infectivity of viral particles from the experiment shown in panel D as evaluated using TZM-bl indicator cells. Lanes are numbered and correspond to the blot above. Statistical comparisons utilized the unpaired t-test, * = p<.05; ** = p<.01.

Saturation of Env on HIV particles could be due to limiting concentrations of a cellular factor that is required for its trafficking or incorporation. We initially investigated a variety of factors involved in cellular recycling pathways as candidates. Depletion of Rab11a or Rab11b did not significantly diminish Env on particles, yet overexpression of the active, GTP-bound form of Rab11a (Rab11a S20V) diminished cellular and particle-associated Env (Fig. S2). This led us to consider the Rab11 Family Interacting Proteins (Rab11-FIPs) as candidates involved in HIV-1 Env trafficking, reasoning that one of the FIPs may have been saturated by Rab11 S20V overexpression. The Rab11-FIPs were originally identified in a yeast 2-hybrid screen using Rab11aS20V as bait [24]. Rab11-FIPs form parallel coiled-coil homodimers and bind to two Rab11a-GTP molecules, creating a heterotetrameric complex that regulates distinct intracellular membrane trafficking events. To examine the potential involvement of the Rab11-FIPs in Env trafficking, we performed shRNA-mediated depletion of each of the described FIPs in HeLa cells. Cells were transfected with NL4-3 proviral DNA and analyzed for particle output, Env content, and infectivity of the released particles. Knockdown efficiency was assessed by real-time PCR, and varied from 65–90% by this assay (Fig. 2A). A striking phenotype was observed in this experiment. Depletion of FIP1C led to markedly diminished particle infectivity, while little effect was seen with depletion of FIP2, FIP3, FIP4, or FIP5 (Fig. 2A). We next verified the knockdown of FIP1C using specific antisera, and examined cellular and viral levels of Env (Fig. 2B). Notably, the cellular levels of gp160 were not altered by FIP1C depletion, while particle-associated Env was markedly lower. Both gp120 and gp41 levels were diminished, suggesting a defect in Env heterotrimer incorporation rather than simply induced shedding of gp120 (Fig. 2B). While depletion of FIP1C removed Env from particles, it had no effect on cellular Env production or on the release of particles (CA bands, Fig. 2B).

The striking effect of FIP1C depletion on Env incorporation suggested to us that it might be an essential cofactor for Env trafficking and particle incorporation. However, to rule out off-target effects, we next restored cellular levels of FIP1C using an shRNA-resistant GFP-tagged FIP1C cDNA. Fig. 2C demonstrates that FIP1C depletion and not transduction with a scrambled shRNA vector depleted cellular FIP1C levels and diminished particle Env incorporation. FIP1C levels were not restored upon expression of the unaltered FIP1C cDNA. In contrast, expression of FIP1C cDNA with silent mutations rendering it resistant to shRNA-mediated depletion was able to fully restore the incorporation of Env onto HIV-1 particles (Fig. 2C, FIP1C cDNA* lanes). Accompanying the restoration of FIP1C function and Env incorporation, the infectivity of the released particles was fully restored (Fig. 2D).

### Rab11-FIP1C mediates Env incorporation in T cell lines

**Figure 3:**
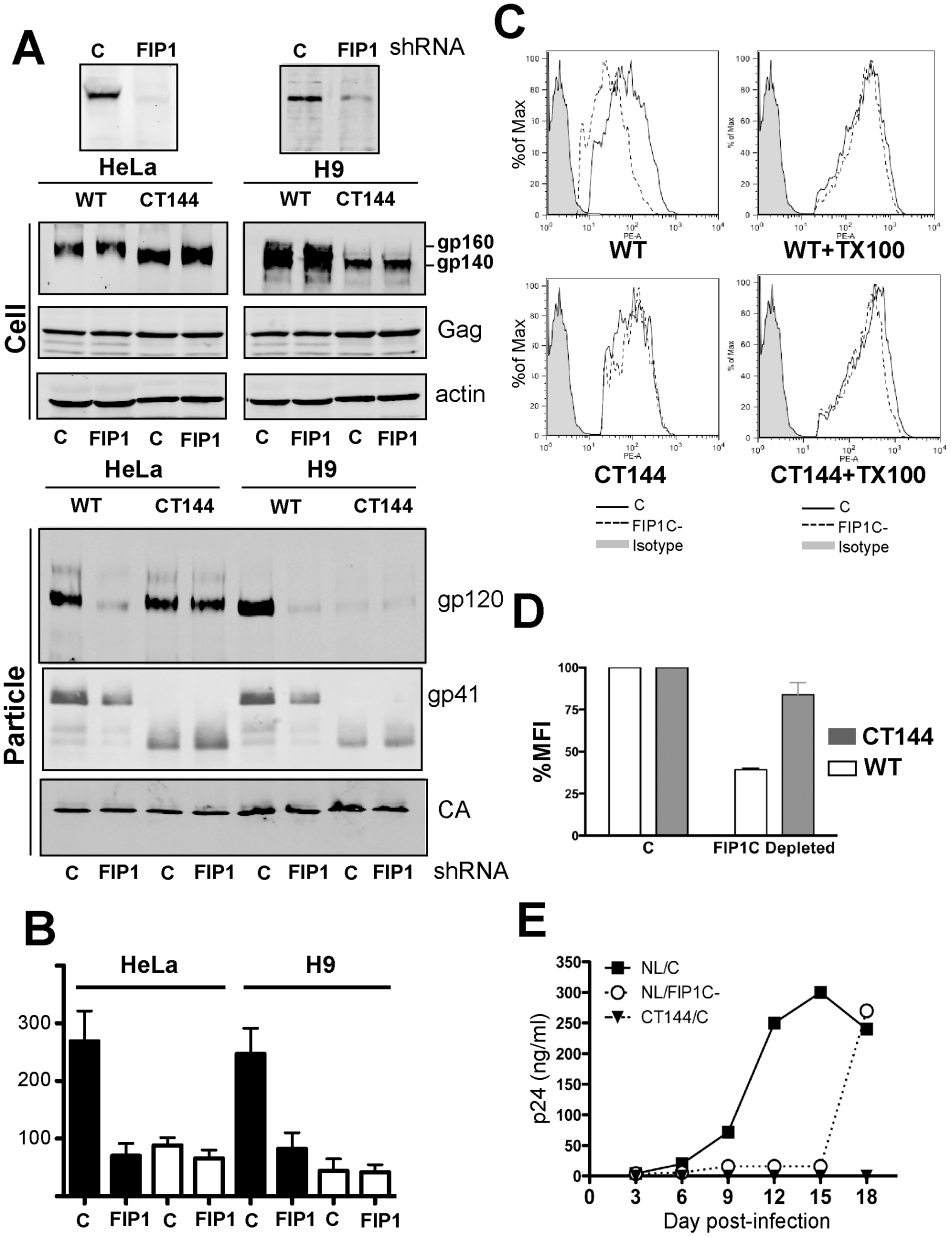
FIP1C depletion alters HIV-1 Env incorporation, cell surface levels, and viral growth in the H9 T cell line. (A) FIP1C depletion was achieved using shRNA in HeLa cells (left) and the H9 T cell line (right). Populations of FIP1C-depleted HeLa and H9 cells were then divided and infected with VSV-pseudotyped NL4-3 or NL4-3delCT144. Released viruses were harvested 2 days after infection (HeLa) or 4 days after infection (H9) and evaluated for protein content by Western blotting. Note that results using specific antiserum used for detection of gp120 and gp160 in cells and particles are shown; gp41 particle blots were probed with a gp41-specific monoclonal antibody. (B) Infectivity of released viral particles from the experiment shown in (A) was measured in the TZM-bl reporter cell line, expressed as infectious units/ng p24. (C) Plasma membrane (left) and total cellular (permeabilized) levels of HIV-1 Env (right) were detected using flow cytometry 4 days after infection with VSV-G- pseudotyped NL4-3 WT or NL4-3delCT144 viruses. The relative level of Env on plasma membrane compared to total Env was calculated and plotted in panel (D) for control and FIP1C-depleted H9 cells. (E) FIP1C-depleted (open circles) and control shRNA-treated H9 cells (filled squares) were infected with VSV-pseudotyped viruses indicated at MOI 0.5. Viral growth was monitored over time by detection of p24 in the culture supernatant. CT144 growth (triangles) shown in control H9 cells.

We next examined the effect of FIP1C depletion on truncated Env using NL4-3delCT144, and extended our analysis to the H9 T cell line. In HeLa cells, depletion of FIP1C markedly diminished Env incorporation in released particles as before (Fig. 3A, WT lanes). CT144 Env incorporation was not altered by depletion of FIP1C (Fig. 3A, CT144 lanes). The effect of FIP1C depletion was even more striking when the experiment was performed in H9 cells (Fig. 3A, H9 particle lanes). CT144 was very poorly incorporated in control-transduced cells in H9 cells, reflecting cytoplasmic tail-dependent particle incorporation of Env as had been reported [23]. The small amount of incorporated CT144 Env was not altered by FIP1C depletion (Fig. 3A). Particle infectivity measured in supernatants from wildtype NL4-3 virus was diminished upon FIP1C depletion in both HeLa and H9 cells, while NL4-3delCT144 particle infectivity was not altered (Fig. 3B). Notably, the effect of FIP1C depletion on HIV-1 Env incorporation was specific for HIV, as no effect on Env incorporation or particle infectivity was observed for viruses pseudotyped with VSV-G or the amphotropic murine leukemia virus (MLV) Env (Fig. S3).

### FIP1C depletion reduces cell surface concentration of HIV-1 Env and inhibits viral replication in T cell lines

FIP1C is involved in directed trafficking of membrane proteins from the endosomal recycling complex to the plasma membrane. We hypothesized that FIP1C depletion would diminish the specific outward trafficking of HIV-1 Env from this compartment. Cell surface Env was measured in control H9 cells versus those depleted of FIP1C. Cell surface gp120 levels were significantly diminished following FIP1C depletion, while total cellular levels of Env were comparable in control and FIP1C-depleted cells (Fig. 3C and 3D, WT). In contrast, no significant effect on cell surface levels of CT144 Env was observed following FIP1C depletion (Fig. 3C and 3D, CT144). We conclude from this that there is a partial depletion of cell surface Env following FIP1C/RCP depletion, and this depletion is itself dependent upon an intact cytoplasmic tail. We next established a population of H9 cells depleted for FIP1C, and infected this population with NL4-3 virus or NL4-3delCT144 at a multiplicity of infection (MOI) of 0.5. No replication of the cytoplasmic tail-truncated virus was observed in H9 cells (Fig. 3E, closed triangles). Wildtype NL4-3 replicated well in H9 cells that had been transduced with control shRNA (filled squares, Fig. 3E). Depletion of FIP1C resulted in markedly delayed and slow replication of NL4-3 (Fig. 3E). At late timepoints, this virus was able to replicate more efficiently, and this was associated with higher levels of FIP1C that developed in the H9 population over time, and not with viral reversion to a FIP1C-independent state (not shown). We concluded from this that FIP1C was essential for Env incorporation and for viral spreading infection.

### Redistribution of FIP1C to the plasma membrane by HIV-1 Env

**Figure 4:**
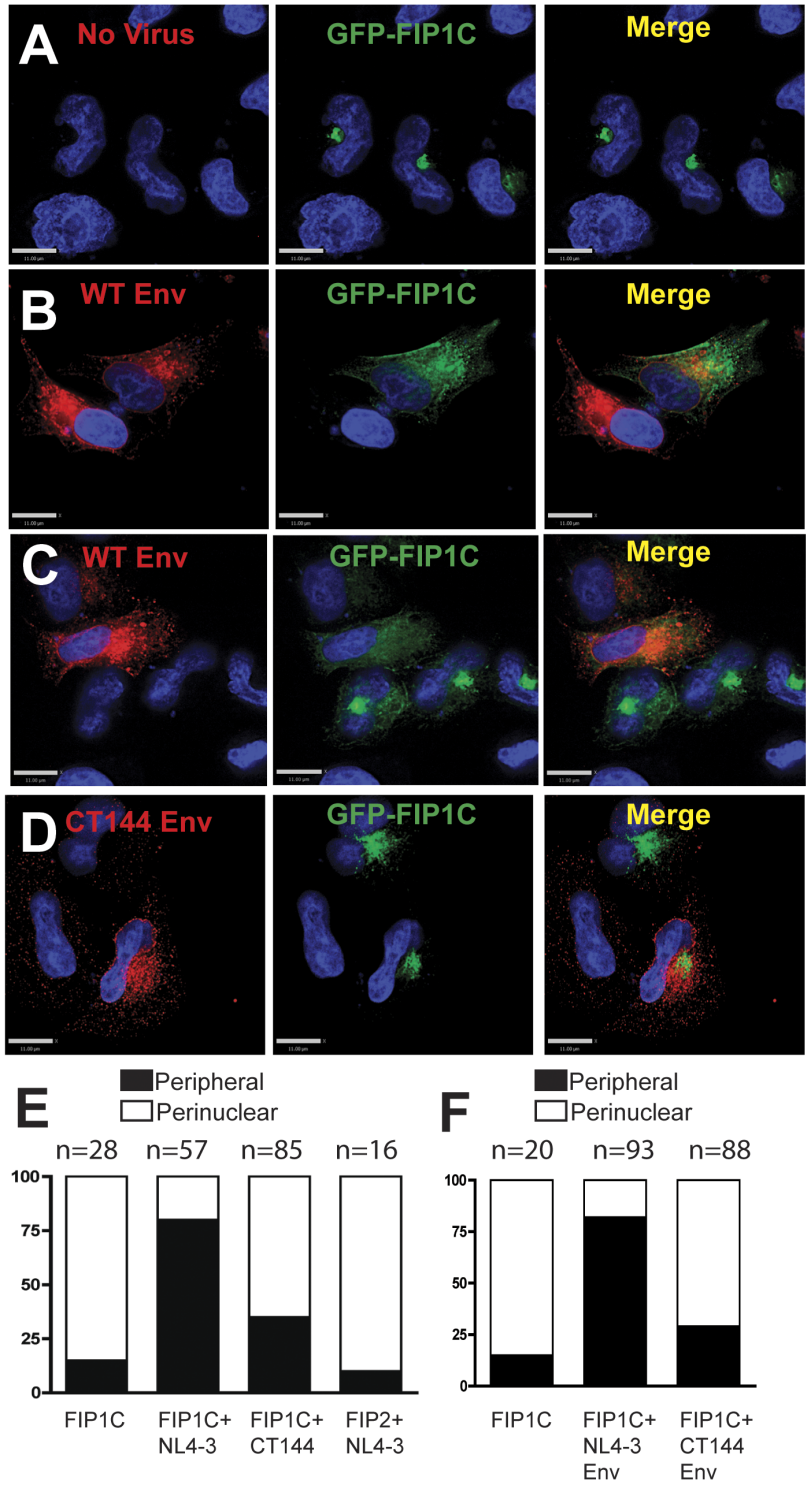
HIV-1 Env redistributes FIP1C to plasma membrane in a gp41 cytoplasmic tail-dependent manner. HeLa cells were transfected with GFP-FIP1C (A–C) and either proviruses NL4-3 WT Env (B,C) or NL4-3delCT144 Env mutant (D). 24 hours after transfection, cells were fixed with 4% PFA and stained for HIV-1 Env using human monoclonal antibody b12. The distribution of GFP-tagged FIP1C, FIP2 and Env was examined using a wide-field deconvolution immunofluorescent microscope. Quantitation of tight perinuclear distribution versus peripherally-distributed GFP-FIP1C was evaluated by blinded observers on a per-cell basis, presented graphically as the % of evaluable cells in each category (E). We also tested if HIV-1 Env protein alone redistributes FIP1C using same method described above and plotted the result in (F).

FIP1C normally resides in a perinuclear compartment that colocalizes strongly with Rab11a [26]. HIV-1 Env is found extensively in intracellular compartments as well as on punctate foci on the plasma membrane, making studies of surface redistribution of Env by cellular factors challenging. However, we reasoned that Env might redistribute GFP-FIP1C in a measurable manner. GFP-FIP1C was found predominantly in a focal perinuclear location in HeLa cells in the absence of HIV-1 Env expression (Fig. 4A). Transfection of NL4-3 provirus led to a marked redistribution of GFP-FIP1C to intracellular vesicles and to the plasma membrane (Fig. 4B). The redistribution of GFP-FIP1C out of the perinuclear compartment was especially evident in fields where cells expressing Env were adjacent to those lacking Env (Fig. 4C). In contrast, transfection of NL4-3delCT144 provirus in general retained the perinuclear distribution of GFP-FIP1C (Fig. 4D). The differences in redistribution of FIP1C out of the perinuclear location were scored by a blinded observer and were significantly different for WT vs. CT144 provirus (Fig. 4E). To determine if this effect was due to Env alone and not to other components of the provirus, the experiment was repeated with expression of WT Env and CT144 Env alone. Again the peripheral redistribution of FIP1C by WT Env was noted, with a much lower level of FIP1C outside of the perinuclear region upon expression of CT144 Env (Fig. 4F). Differences in FIP1C distribution between full-length and CT144 Env were statistically significant (p<0.001 by chi squared test). These results suggest a CT-dependent outward movement of FIP1C.

### Mapping the interaction between FIP1C and Rab14

**Figure 5:**
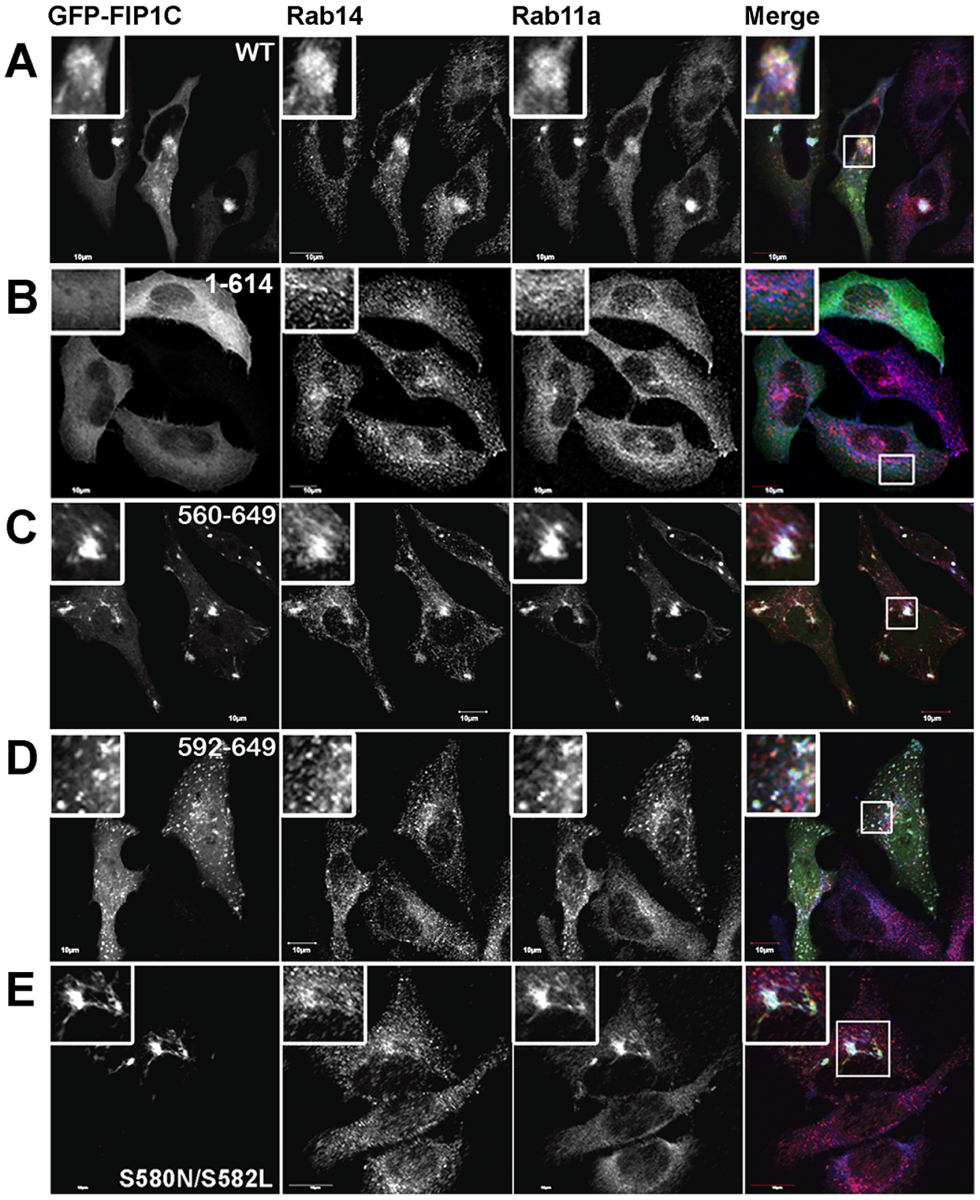
GFP-FIP1C-wildtype and FIP1C truncation mutants demonstrate distinct localization with Rab14 and Rab11a. HeLa cells were transfected with (A)GFP- FIP1C wild-type (WT; 1–649), (B) C-terminally truncated GFP-FIP1C (1-614), lacking the Rab11 binding domain (RBD), (C) N-terminally truncated GFP-FIP1C (560–649), (D) GFP-FIP1C (592–649), or (E) FIP1C(560–649)(S580N/S582L). Cells were fixed and stained for endogenous Rab14 (red) and Rab11a (blue).

While FIP1C was critical to Env incorporation onto particles, depletion of Rab11 itself did not significantly alter Env incorporation (discussed further below). To reconcile how a Rab11a-binding protein acting as an adaptor could modulate Env incorporation in a Rab11a-independent manner, we investigated the involvement of Rab14 in Env incorporation. Rab14 has recently been identified as a Rab11-FIP binding protein [27]. These investigators had suggested that Rab14 might interact with the Rab binding domain of multiple Rab11-FIP proteins. We therefore sought to investigate the association of Rab14 with different Rab11-FIPs using a split-ubiquitin yeast two-hybrid assay as a method for assessing protein interactions. While we did confirm that Rab14 interacted with FIP1C as well as FIP1B, we did not find any interaction with FIP2 (Supplemental Table S1 in Text S1). All three Rab11-FIP proteins interacted with Rab11a, but none interacted with Rab8a. Given these results, we next evaluated whether Rab14 associated *in situ* with FIP1C. Fig. 5A demonstrates that GFP-FIP1C colocalized extensively with both Rab11a and Rab14 in a concentrated compartment representing the ERC. However, FIP1C(1–614), which lacks a Rab11a binding domain was distributed throughout the cytosol rather than concentrated in the ERC, and did not colocalize with either Rab11a or Rab14 (Fig. 5B). The carboxyl terminal truncation Rab11-FIP1C(560–649) caused a marked concentration of both Rab11a and Rab14 with the dominant negative construct (Fig. 5C). Nevertheless, while GFP- FIP1C(592–649), which retains the Rab11-binding domain, did colocalize extensively with Rab11a, the localization with Rab14 was markedly attenuated (Fig. 5D). These results suggested that Rab14 may not be associating with the Rab11-binding domain as suggested by Kelly at al. (2010). Since Kelly et al. (2010) had suggested that Rab14 associated with other Rab11-FIP proteins through their conserved Rab11-binding domains, we next examined the association of Rab14 with the dominant negative trafficking mutant GFP-FIP2(129–512). While this mutant strongly concentrated Rab11a, it had no effect on Rab14 distribution (Fig. S4). Similarly, we did not observe any effects of a carboxyl terminal fragment of FIP5 on Rab14 distribution, although it did strongly concentrate Rab11a (data not shown). These immunofluorescence studies, combined with yeast two-hybrid results, indicated that Rab14 is not interacting with the Rab binding domain of Rab11-FIP1C, since this helix is strongly conserved in all FIP proteins.

To evaluate further the binding requirements for Rab14 with FIP1C, we compared the sequences of FIP1C and FIP2 in their carboxyl termini and performed directed mutagenesis of candidate residues that were different between the two Rab11-FIPs. We mutated two residues from FIP1C to those matching the sequence in FIP2 to create FIP1C(S580N/S582L). Fig. 5E shows that this double mutation in the context of GFP-FIP1C(560–649) elicited a loss of colocalization with Rab14, while colocalization with Rab11a was unaffected. These results demonstrate that the region proximal to the Rab11-binding domain in FIP1C is responsible for association with Rab14, separate from the Rab11a association requirements. We next confirmed direct binding of WT GFP-FIP1C but not GFP-FIP1C(S580N/S582L) with Rab14 through co-immunoprecipitation studies (Fig. S5). Having mapped the Rab14 binding domain on FIP1C, we next asked if the Rab14-FIP1C interaction was responsible for the FIP1C-dependent incorporation of Env onto HIV-1 particles.

### Rab14 mediates HIV-1 Env incorporation onto HIV-1 particles

**Figure 6:**
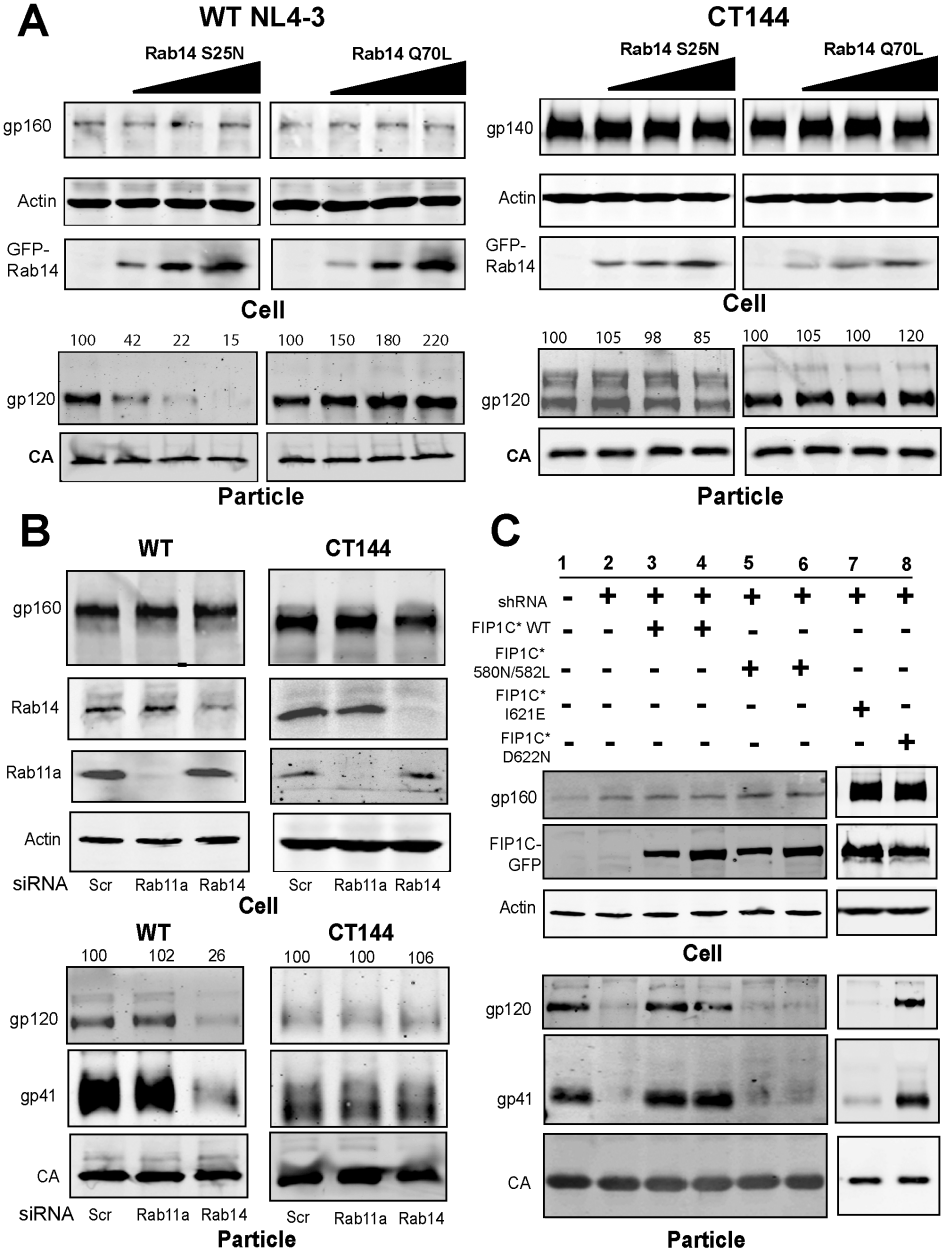
Rab14 interaction with FIP1C is required for HIV-1 Env incorporation. (A) HeLa cells were transfected with proviral plasmid pNL4-3 or NL4-3 CT144 and either a dominant negative form of Rab14 (Rab14S25N) or a constitutively active form of Rab14 (Rab14Q70L). 48 hours after transfection, cellular and particle content of HIV-1 Env was examined by Western blot. Numbers above Env blots represent densitometry values with leftmost lanes set to 100. (B) shRNA-mediated depletion of Rab11 or Rab14 was performed in HeLa cells. Following selection in puromycin, cells were transfected with either pNL4-3 or pNL4-3 CT144 plasmid. Cellular and particle-associated Env was detected by immunoblotting 48 hours following transfection. (C) shRNA-resistant GFP-FIP1C* WT and GFP-FIP1C* (S580N/S582L) were transfected together with pNL4-3 in control HeLa cells or FIP1C-depleted HeLa cells as in the repletion experiment described in Figure 2. Input FIP1C* plasmid levels were 2 µg in lanes 3, 5, 7, and 8 and increased to 4 µg in lanes 4 and 6. In a separate experiment the ability of Rab11 binding mutant GFP-FIP1C* I62E and GFP-FIP1C* D622N to rescue Env particle incorporation in FIP1C-depleted HeLa cells (lanes 7 and 8). Note that gp160 and gp120 were detected with polyclonal antiserum; gp41 particle blots were probed with a monoclonal specific for gp41.

Based on the interaction between FIP1C and Rab14, we considered the possibility that Rab14 is involved in HIV-1 Env trafficking and incorporation onto HIV particles. We first examined the effects of dominant-negative (S25N) and constitutively-active (Q70L) forms of Rab14 on incorporation of HIV-1 Env. Remarkably, dominant-negative Rab14(S25N) diminished the incorporation of Env onto HIV-1 particles in a dose-dependent fashion (Fig. 6A). Constitutively-active Rab14(Q70L) expression had the opposite effect, and resulted in an enhanced level of particle-incorporated Env (Fig. 6A, Rab14Q70L). These results supported an important role for Rab14 in HIV-1 Env incorporation. In the absence of FIP1C, the enhancement of particle-incorporated Env by Rab14(Q70L) was not observed (Fig. S6A). Consistent with our prior findings of CT-dependence, Rab14 constitutively-active and dominant-negative constructs had no effect on incorporation of CT144 Env onto particles (Fig. 6A, rightmost panels). Combined with the interaction mapping data above, these data strongly suggest that an interaction between FIP1C and Rab14 is required for incorporation of full-length HIV-1 Env onto particles.

To evaluate further the role of Rab14 on Env incorporation, we depleted Rab14 or Rab11a in HeLa cells using shRNA and compared the effects on Env incorporation. Rab14 depletion, but not Rab11a depletion, diminished HIV-1 Env incorporation (Fig. 6B). The effect of Rab14 depletion was specific for HIV-1 Env, as it had no effect on pseudotyping of HIV particles with VSV-G or amphotropic MLV Env (Fig. S6B). To further examine the specificity of the Rab14-FIP1C interaction in mediating HIV Env incorporation, we utilized the interaction domain mapping data outlined above, and asked if FIP1C(S580N/S582L), which does not interact with Rab14, could rescue Env incorporation in FIP1C-depleted HeLa cells. Strikingly, shRNA-resistant wildtype FIP1C rescued Env incorporation as before, while the 580/582 double mutant was unable to rescue Env onto particles (Fig. 6C, compare lanes 3 and 4 to lanes 5 and 6). This indicates that Rab14 interaction is required for rescue of Env particle incorporation. We then introduced mutations in the Rab11 binding domain that have been shown to eliminate Rab11a binding and disrupt membrane association (FIP1C(I621E)) or that eliminate Rab4 interactions without disrupting membrane interactions (FIP1C(D622N)) [28], placing these both in the context of the shRNA-resistant FIP1C construct. When compared head-to-head, FIP1C(I621E) was not able to rescue Env particle incorporation (Fig. 6C, lane 7) while FIP1C(D622N) was competent for rescue (Fig. 6C, lane 8). We interpret this to mean that mislocalized I621E does not reach the ERC, and subsequently cannot direct outward sorting of Env. It is unclear whether this means that normally Rab11 binding is required for the initial ERC localization of FIP1C, followed by Rab14-directed outward sorting, or if the mislocalization and lack of rescue of Env incorporation is reflective of other defects elicited by this mutation. The fact that knockdown of Rab11a or Rab11b did not alter Env incorporation (Fig. S2A) suggests the latter interpretation, but further work will be needed to define this. Taken together, our data support a model in which a specific Rab14/FIP1C complex mediates the intracellular trafficking and particle incorporation of the HIV-1 Env glycoprotein complex.

### FIP1C mediates Env incorporation in primary human macrophages

**Figure 7:**
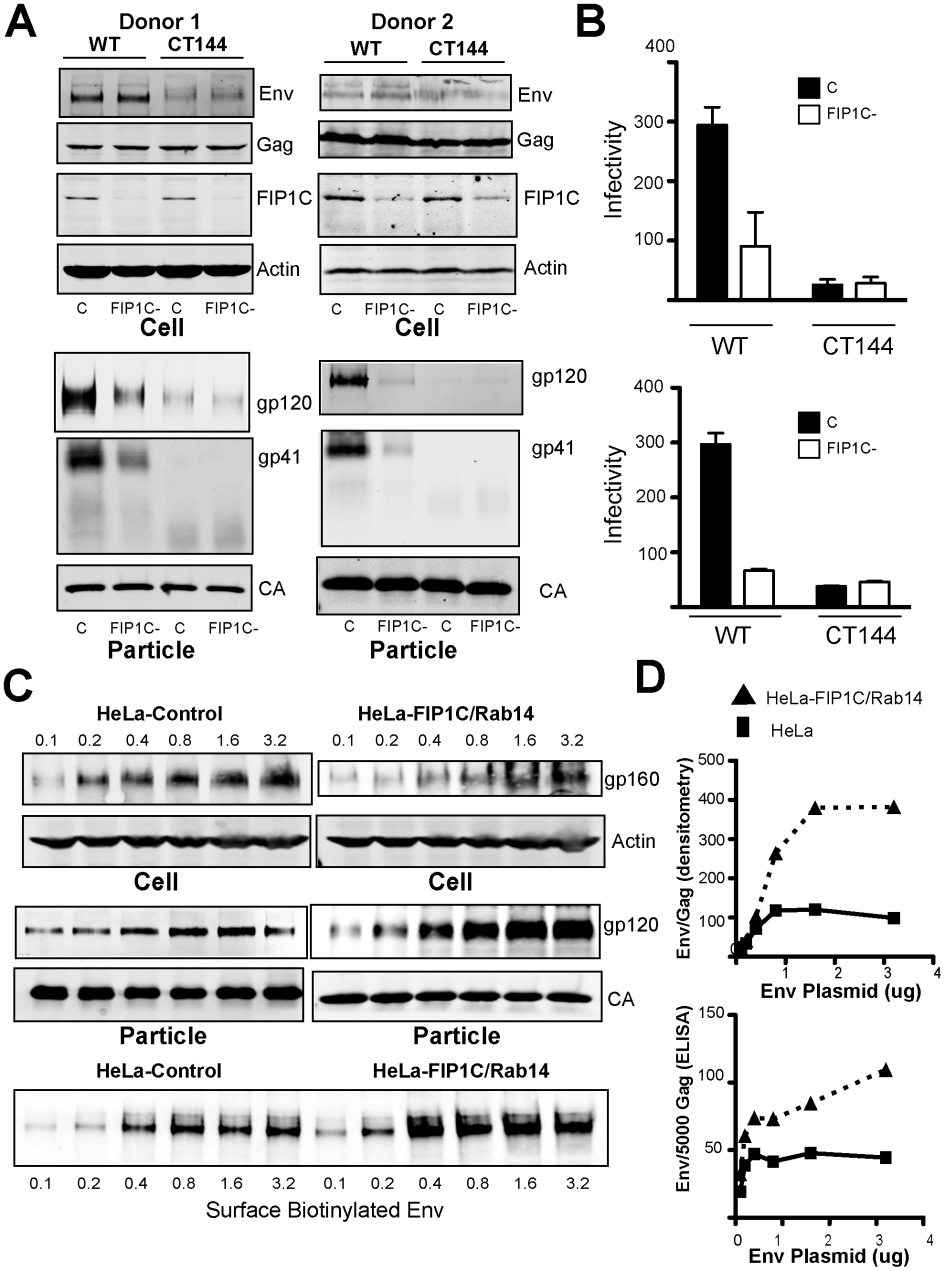
FIP1C is required for Env incorporation in MDMs and enhances Env content of HIV-1 particles when overexpressed with Rab14. (A) MDMs were prepared from peripheral blood of healthy donors and depleted of FIP1C using two administrations of siRNA. MDMs were then infected with VSV-G-pseudotyped NL4-3 or NL4-3delCT144. Cell lysates and purified particles were examined 8 days following infection. Shown are Western blot results from two donors. (B) Particle infectivity from the experiments described above was measured in TZM-bl reporter cells as before. (C) Parental HeLa T-REx cells (Invitrogen) and HeLa-T-REx cells stably transfected with plasmids for inducible expression of FIP1C and Rab14Q70L were transfected with a constant amount of Gag expression plasmid and increasing amounts of NL4-3 Env plasmid (numbers above gp160 blot represent µg of input plasmid). Cell lysates and purified particles were examined by Western blotting. In these experiments, virus lanes were loaded with equal amounts of p24 as measured by ELISA. The bottom Env immunoblot represents cell surface Env as determined by biotinylation and precipitation with streptavidin beads, using the same input Gag and Env DNA as above. (D) Env and Gag content of released viral particles was quantified by densitometry using the LiCor instrument and plotted as background-subtracted Env pixel intensity to Gag pixel intensity (left) and by p24 and gp120 antigen-capture ELISA (right). The number of Env molecules per particle was calculated as an approximation, assuming that 5000 CA molecules are present in one HIV-1 virion.

Cytoplasmic tail-dependent incorporation of HIV-1 Env onto HIV particles is not limited to T cells, but also has been demonstrated in monocyte-derived macrophages (MDMs) [23]. We therefore sought to determine if FIP1C-dependent Env incorporation was also present in MDMs. Using an efficient siRNA method we recently established in MDMs [29], we depleted FIP1C in primary MDMs from two donors, then infected the cells with VSV-G-pseudotyped wildtype NL4-3 or NL4-3delCT144. As expected, incorporation of gp120 and truncated gp41 on particles from NL4-3delCT144-infected cells was markedly lower than wildtype NL4-3 (Fig. 7A). Depletion of FIP1C in MDMs significantly diminished gp41 and gp120 particle incorporation (Fig. 7A) and infectivity (Fig. 7B). These data indicate that FIP1C is required for the CT-dependent incorporation of Env onto HIV particles in MDMs as it is in T cell lines and HeLa cells.

### FIP1C and Rab14 overexpression enhances Env incorporation in HeLa cells

Our initial rationale for searching for cellular trafficking factors involved in Env incorporation was the fact that limitations to Env particle incorporation were not related to the amount of Env produced in infected or transfected cells (Fig. 1). After identifying the involvement of FIP1C and Rab14 in Env incorporation, we wished to determine if overexpression of FIP1C and Rab14 would therefore overcome this limitation and significantly enhance Env incorporation. We created a cell line that inducibly overexpressed FIP1C and Rab14Q70L, and repeated the titration of Env expression in the presence of a constant amount of cellular Gag. Remarkably, the expression of FIP1C and Rab14Q70L enhanced Env incorporation by 2–3 fold (Fig. 7C). Cell surface levels of Env were also increased upon overexpression of FIP1C and Rab14Q70L as assessed by a surface biotinylation assay (Fig. 7C, bottom). The increase in Env incorporation on particles released from this cell line was quantified by densitometric evaluation of Env/Gag ratios and by determination of Env and Gag particle content by ELISA (Fig. 7D). We conclude that FIP1C and Rab14 are limiting factors for Env particle incorporation in HeLa cells.

## Discussion

Retroviral envelope proteins are incorporated onto developing viral particles by a process that remains incompletely understood. Pseudotyping of viruses or virus-like particles (VLPs) with envelope glycoproteins of foreign viruses has revealed both specific and non-specific mechanisms of Env incorporation. HIV-1 can form infectious particles with Env proteins from a wide variety of retroviral subfamilies, including alpha-, beta-, gamma-, and delta-, and even spumaretroviruses [30], [31], [32], [33], [34] (reviewed in [35]). This promiscuity for pseudotyping extends to Env glycoproteins from other viral families, including coronaviruses [36], paramyxoviruses [37], filoviruses [38], rhabdoviruses [39], and others. Promiscuity of Env incorporation would argue either for a lack of specificity of Env incorporation, or for a common mechanism that is shared by these diverse viruses for Env delivery and incorporation. In contrast, HIV-1 Env itself is quite selective in its ability to pseudotype other retrovirus particles. HIV-1 Env with a full length CT is unable to pseudotype MLV vectors, although truncation of Env CT allows MLV pseudotyping [40]. HIV-1 mutants with point mutations in MA fail to incorporate full-length HIV-1 Env, while truncation of CT allows Env incorporation [41], [42]. Although direct interactions between Env CT and MA have been reported in vitro for HIV and SIV [43], [44], a direct interaction has been difficult to substantiate in cell-based assays. Thus there is selectivity and specificity of particle incorporation that is conferred by the full length HIV-1 Env CT, while the mechanism has not yet been fully explained. The specificity of HIV-1 Env incorporation could be due to direct interactions with MA, to indirect Gag-Env interactions mediated by an intermediate host protein, or could be explained by cotrafficking to a common site of assembly as outlined by Jorgenson and colleagues [45]. Our data support a requirement for specific outward Env trafficking from the ERC mediated by FIP1C and Rab14.

Functionally, the Rab11-FIPs play important roles in the recycling of cargo from the recycling system to the cell surface. Rip11/Rab11-FIP5 is required for the transport of GLUT4 vesicles to the cell surface following insulin treatment [46]. FIP2 regulates the recycling of a number of plasma membrane proteins, including aquaporin-2 and CXCR2, to the cell surface [47], [48]. FIP2 has also been implicated in trafficking and assembly of respiratory syncytial virus, providing prior evidence that viruses may usurp the FIP-mediated transport mechanisms to deliver their cargo to the site of assembly [49]. Most significant for this report is the ability of FIP1C to regulate the recycling of α5β1 integrin and EGFR1 to the cell surface [50].

We identified FIP1C and its binding partner Rab14 as essential components determining the incorporation of full-length but not truncated Env onto HIV-1 particles. Rab11-FIP1 was first identified in 2001 through a yeast 2-hybrid screen employing dominant active Rab11a as bait [24]. Rab11a and Rab11-FIPs localize to the endosomal recycling complex and to the apical recycling endosome in polarized cells, where they regulate sorting and plasma membrane recycling [25], [26]. There are five members of this family that have been divided into two classes based upon the presence of specific motifs including C2 domains (class 1 members FIP1B, FIP1C, FIP2, and Rip11/FIP5) and EF hands (class 2 members FIP3 and FIP4) [25], [51]. All family members possess a carboxyl-terminal alpha-helical Rab11-binding domain (RBD). The RBD of the FIPs forms a parallel coiled-coil homodimer that interacts with switch 1 and switch 2 regions of the GTP-bound form of Rab11a, resulting in a heterotetrameric complex of two FIPs and two Rab proteins [52], [53], [54], [55]. While a previous investigation has suggested that the class I FIPs are capable of forming complexes with the Rab14 GTPase [27], our findings suggest that Rab14 interacts specifically with FIP1C through a region separable from the Rab11 binding domain. These results indicate that FIP1C has multiple binding domains for small GTPases, as previously demonstrated for Rabaptin-5 (Rab5 and Rab4) and FIP3 (Arf6 and Rab11) [29], [56]. Our studies demonstrate the discrete point mutations in FIP1C proximal to the RBD can disrupt Rab14 binding without affecting Rab11a association. Similarly, we could not demonstrate any association between Rab14 and FIP2 or FIP5 in either split ubiquitin yeast two-hybrid assays or in immunofluorescence studies. The difference between our studies and those of Kelly, et al. (2010) could be due to differences in the cell systems examined, but it seems more likely that the discrepancy stems from the antibodies used to detect Rab14. The Aviva Rab14 antibody we have used here has no cross reactivity with either Rab11a or Rab7. In contrast, we have found that the antibody used by Kelly, et al. [27] has prominent cross-reactivity with recombinant Rab11a and Rab7 (data not shown). In any case, the fact that the S580N/S582L FIP1C dual mutant was unable to rescue Env particle incorporation confirms that it is the Rab14/FIP1C interaction rather than the interaction of Rab11a with FIP1C that defines the trafficking complex for HIV Env. In support of this, we found that the GTP-bound form of Rab14(Q70L) actually enhanced the amount of particle-associated Env, while dominant-negative Rab14(S25N) diminished Env incorporation in a dose-dependent fashion. Similar experiments with dominant-negative and constitutively-active Rab11a did not demonstrate this effect. In fact, the constitutively-active Rab11a(S20V) construct diminished Env incorporation, perhaps through competition for FIP1C binding. Together, our results support a complex of GTP-bound Rab14 and FIP1C as essential components mediating the transport of HIV-1 Env to the particle assembly site.

**Figure 8:**
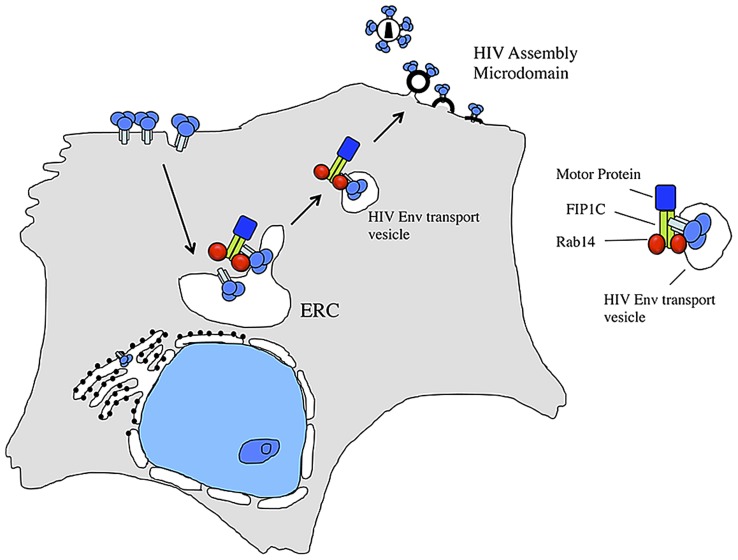
Model for outward sorting of HIV Env by the FIP1C-Rab14 complex. ERC = endosomal recycling compartment.

Our data support a model of directed outward trafficking of HIV-1 Env to a plasma membrane microdomain for specific incorporation onto the lipid envelope of budding virions. We suggest that FIP1C and Rab14 are required for this directed trafficking from the ERC to the site of particle assembly and budding, as depicted in Fig. 8. This model predicts that cellular levels of specific trafficking factors may limit the number of Env trimers incorporated onto particles. In support of this idea, we were able to modestly increase the amount of Env on HIV particles by overexpression of constitutively-active Rab14. We propose that the delivery of Env trimers to the cell surface is required but not sufficient for particle incorporation for wildtype viruses bearing intact CTs. Truncated Env constructs such as CT144 reach the cell surface of permissive cells and may be acquired passively, explaining the lack of saturation of truncated Env shown in Fig. 1 of this report. However, T cells are not permissive for incorporation of truncated Env [23]. Therefore the nature of the assembly microdomain in which assembly occurs may differ between cell types such as HeLa cells and H9 cells, with exclusion of truncated Env from this microdomain in H9 cells and lack of exclusion in HeLa. Active trafficking of full length Env, directed by Rab14 and FIP1C, is required for Env delivery and particle incorporation in both cell types. The tail dependence of this phenotype predicts that specific motifs in the CT will be engaged with a FIP1C/Rab14 complex in the outward trafficking of Env. Future work will focus on the identification of these motifs and the interacting domains within FIP1C. We note that there are numerous polymorphisms in FIP1C alleles reported in the NCBI database, some of which could be damaging to its function. It will be important in future studies to examine the potential effect of polymorphisms in FIP1C on HIV acquisition or disease progression.

In conclusion, we have identified for the first time a complex of Rab14 and Rab11-FIP1C with a biologically-relevant cargo. The Rab14/FIP1C complex directs HIV-1 Env to the plasma membrane assembly site for incorporation onto developing particles in a manner that depends upon the long cytoplasmic tail of Env. It will be of great interest to define other components of this trafficking complex, such as motor protein(s) involved in the outward trafficking of Env, and to extend these findings to the assembly processes of other retroviruses as well as more distant families of viruses.

## Materials and Methods

### Ethics statement

Peripheral blood was obtained from healthy volunteer donors according to a protocol approved by the Emory University Institutional Review Board (IRB). Written informed consent was obtained from donors, and samples were de-identified prior to handling by laboratory personnel.

### Cells and media

HeLa and TZM-bl cells [57]) were maintained in DMEM containing 10% FBS and antibiotics. The H9 T cell line (ATCC HTB-176) were cultured in RPMI medium 1640 supplemented with 10% FBS, 2 mM Glutamine, and antibiotics. MDMs were prepared from human peripheral blood as described previously [29]. HeLa T-REX cells for inducible expression of FIP1C and Rab14 were created using the T-REx system and vector pCDNA5/TO (Invitrogen). Cells were maintained in tetracycline-free media and induction carried out with 1 µg/ml doxycycline.

### Viruses, plasmids, and lentiviral vectors

Vesicular stomatitis virus G glycoprotein (VSV-G)-pseudotyped HIV-NL4.3 and HIV-CT144 virus stocks were generated by cotransfecting 293T cells with pNL4.3 or pNL4.3CT144 and the VSV-G expression plasmid pHCMV-G. Viruses were harvested from transfected 293T supernatants 48 hours post-transfection, filter-sterilized, and assayed with TZM-bl indicator cells for infectivity assessment. Env expression plasmids pIIINL4env and pIIINL4envCTdel-144 were gifts kindly provided by Dr. Eric Freed [23]. Vesicular stomatitis virus glycoprotein (VSV-G) expressing plasmid pHCMV-G was provided by J. Burns [58]. Amphotropic MLV Env expression vector pCL-Ampho was from Inder Verma [56]. Rab11a expression plasmids and FIP1C expression plasmids have been previously described [24], [26], [59]. FIP1C-specific shRNA constructs and methods, siRNA sequences, and real-time PCR reagents are described in Supplemental Experimental Procedures. Silencing of FIP1C-expression in MDMs was carried out by transfecting FIP1C-specific siRNA (Dharmacon) using the N-TER nanoparticle siRNA transfection system (Sigma) as previously described [29].

### Antibodies and immunostaining reagents

Rabbit polyclonal antibody against FIP1C was obtained from Sigma. Goat polyclonal antibody against HIV-1 gp120 and gp160 (used in Western blotting) was AHP2204 from AbD Serotec (Oxford, UK). Human anti-gp120 antibody for immunofluorescence experiments was IgG1 b12; synthesized from recombinant cDNA by the laboratory of James Crowe (Vanderbilt University). Antibody used for immunoblotting of gp41 was murine monoclonal 5009 from BTI research reagents (Columbia, MD). HIV Gag detection was performed with either rabbit anti-p17 polyclonal, mouse anti-p24 monoclonal CA-183 (provided by Bruce Chesebro and Kathy Wehrly through the NIH AIDS Research and Reference Reagent Program), or mouse anti-p24-FITC (KC57-FITC) obtained from Beckman Coulter (Fullerton, CA, USA). Anti-VSV-G antibody was from Sigma (V5507), and anti-amphotropic MLV goat antiserum (679) was obtained from Chris Aiken (Vanderbilt). Rabbit anti-Rab14 antibody was obtained from Aviva Systems Biology. Mouse monoclonal anti-Rab11a (8H10) was described previously by the Goldenring laboratory [60], who also provided rabbit anti-Rab11b polyclonal antiserum (VU76). Alexa Fluor goat anti-mouse and Alexa Fluor goat anti-rabbit secondary antibodies, as well as the DAPI nucleic acid stain were obtained from Molecular Probes (Eugene, OR, USA). IRDye goat anti-mouse and IRDye goat anti-rabbit secondary antibodies used for Western blots were obtained from Li-cor Biosciences (Lincoln, NE, USA).

### Image acquisition and analysis

Images of GFP-FIP1C and Env distribution were obtained with a Nikon TE2000-U spinning disc confocal fluorescence microscope with automated stage and Hamamatsu EM-CCD camera developed by Improvision under the control of the Volocity software, or with a DeltaVision imaging system developed by Applied Precision. The system was equipped with an Olympus IX70 microscope and a CoolSnap HQ2 digital camera under the control of the softWoRx software. Imaging processing and deconvolution was performed using softWoRx 3.7.0. Colocalization measurements were quantified with the colocalization function of Volocity 5.2.1. For immunofluorescence experiments, HeLa cells were washed with PBS and fixed in 4% paraformaldehyde for 12 minutes at RT. After fixation, cells were extensively washed including an overnight wash at 4 degree. Cells were then permeabilized for 10 minutes with 0.2% Triton X-100 and block in Dako blocking buffer for 30 minutes. Primary and secondary antibodies were diluted in Dako antibody diluent to appropriate concentrations. DAPI was used to stain the nuclei of the cells. The coverslips were mounted in Gelvatol overnight and examined directly the next day. Images of endogenous Rab11 and Rab14 together with GFP-FIP1C were obtained using an Olympus Fluoview confocal microscope; details of immunofluorescence staining protocols and antibodies for these experiments are provided in Supplemental Experimental Procedures.

### Flow cytometry for HIV-1 Env

HeLa and H9 surface staining was performed with human monoclonal anti-gp120 antiserum at final concentration of 0.1 ug/ml in PBS with 2% BSA and a second PE-conjugated anti-human antibody at 0.02 ug/ml. Mouse anti-p24-FITC (KC57-FITC, Beckman-Coulter) was employed following permeabilization to allow gating on the infected population. HeLa cells were harvested at 48 hours post-infection in this analysis, while H9 cells were harvested at day 4 post-infection. Analysis was performed on a FACSCanto flow cytometer (BD Biosciences) and using FlowJo software (Treestar, Inc).

### Virus release assays and infectivity assays

At 48 hours post transfection, virion- containing culture supernatants were harvested, clarified by low speed centrifugation and filtered (0.2 µm). Infectious virus release was determined by inoculating TZM-bl indicator cells, plated the previous day in 12 well plates at 1×10^5^ cells/well, with 400 µl of serially diluted supernatants. At 48 hours after infection, cells were fixed and stained with X-gal. Blue cells were counted and infectivity was calculated as blue cell numbers per nanogram of p24 inoculation. The remainder of the virion containing supernatant (750 µl) was layered onto 200 µl of 20% sucrose in PBS and centrifuged at 20,000 g for 2 hours at 4°C. Virion pellets, and corresponding virion producing cells were dissolved in SDS PAGE loading buffer. Virion and cell lysates were separated on 10% polyacrylamide gels and subjected to Western blotting.

## Supporting Information

Figure S1 **Env vs. Env + Gag pelleting.** In order to confirm that overexpression of Env was not leading to pelletable Env in the absence of Gag, we performed a titration of Env expression in HeLa cells without (left) or with (right) a constant amount of Gag expression. Supernatant materials were pelleted through 20% sucrose cushions and analyzed by Western blotting for the proteins shown.
(TIF)

Figure S2 **Recycling factor involvement in Env incorporation.** A) Rab11a or Rab11b depletion does not alter Env incorporation. shRNA-mediated depletion of Rab11b (center lanes) or Rab11a (right lanes) was performed in HeLa cells, followed by expression of NL4-3 provirus. Cell lysates (left lanes) and pelleted viral particles (right) were analyzed by Western blotting for the indicated proteins. B) Overexpression of Rab11aS20V depletes particle-associated Env and leads to degradation of cellular Env. An expression plasmid for Rab11a S20V was titrated in HeLa cells co-expressing NL4-3 (left) or NL CT144 (right). Cellular Env levels and particle-associated Env were evaluated by Western blot. Full-length Env was depleted by S20V, while truncated Env was unaffected.
(TIF)

Figure S3 **Effect of Rab11-FIP1C/RCP depletion on HIV particle pseudotyping with amphotropic MLV and VSV-G.** (A) The effect of FIP1C/RCP depletion on amphotropic MLV Env (middle panel) and VSV-G protein (lower panel) incorporation onto HIV-1 particles was examined and compared with effects on gp120 particle incorporation (top). (B) Infectivity of pseudotyped viruses from (A) was measured using TZM-bl reporter cells; units are infected cells/ng p24 input.
(TIF)

Figure S4 **Rab14 does not associate with Rab11-FIP2.** HeLa cells were transfected with either (A) wild type GFP-Rab11-FIP2 or (B) GFP-FIP2 (129–512), designated FIP2(ΔC2). Cells were fixed and then immunostained for endogenous Rab14 and endogenous Rab11a. Inserts show higher magnification regions. Note that FIP2(ΔC2) strongly concentrated Rab11a in association with the EGFP-chimera, but had no effect on Rab14 distribution.
(TIF)

Figure S5 **Co-immunoprecipitation of FIP1C with Rab14.** HeLa Cells were transfected with EGFP-FIP1C WT or EGFP-FIP1C (S580N/S582L) and EGFP-Rab14. Input protein content is shown on left. IP was performed using FIP1C-specific antisera, followed by immunoblotting for the proteins indicated on the right. Endogenous Rab11a is shown.
(TIF)

Figure S6 **Rab14 enhancement of HIV-1 Env incorporation requires FIP1C and Rab14 depletion does not alter incorporation of MLV or VSV-G Env**. A) Titration of Rab14Q70L in HeLa cells with normal FIP1C levels (Scr shRNA lanes) or in cells depleted of FIP1C (FIP1C shRNA lanes). Note that gp120 and gp160 blots were probed with goat anti-gp120/gp160 antisera, while gp41 was probed with murine monoclonal anti-gp41 antibody. B) Hela cells were transfected with NL4-3 or with NL4-3deltaEnv and expression constructs pHCMV-G (for VSV-G) or pCL-Ampho (MLV Env). Depletion of Rab14 was performed using shRNA in the indicated lanes. Cellular and particle Env content was assessed by immunoblotting with specific antisera for HIV gp120/160, VSV-G, or amphotropic MLV Env.
(TIF)

Text S1 1) Table S1: Split-ubiquitin yeast two-hybrid assay of Rab11-FIP protein interaction with Rab proteins. Split ubiquitin assays were performed between CCW vectors with Rab11-FIP proteins and DSL vectors with Rab proteins. Results were consistent over three separate experiments. 2) Supplemental Experimental Procedures.
(DOC)
