# Supplementary figures and images for: Rab11-FIP1C and Rab14 Direct Plasma Membrane Sorting and Particle Incorporation of the HIV-1 Envelope Glycoprotein Complex
Source: PLoS Pathog. 2013 Apr 4;9(4):e1003278. doi: 10.1371/journal.ppat.1003278 (PMC3616983; doi:10.1371/journal.ppat.1003278)

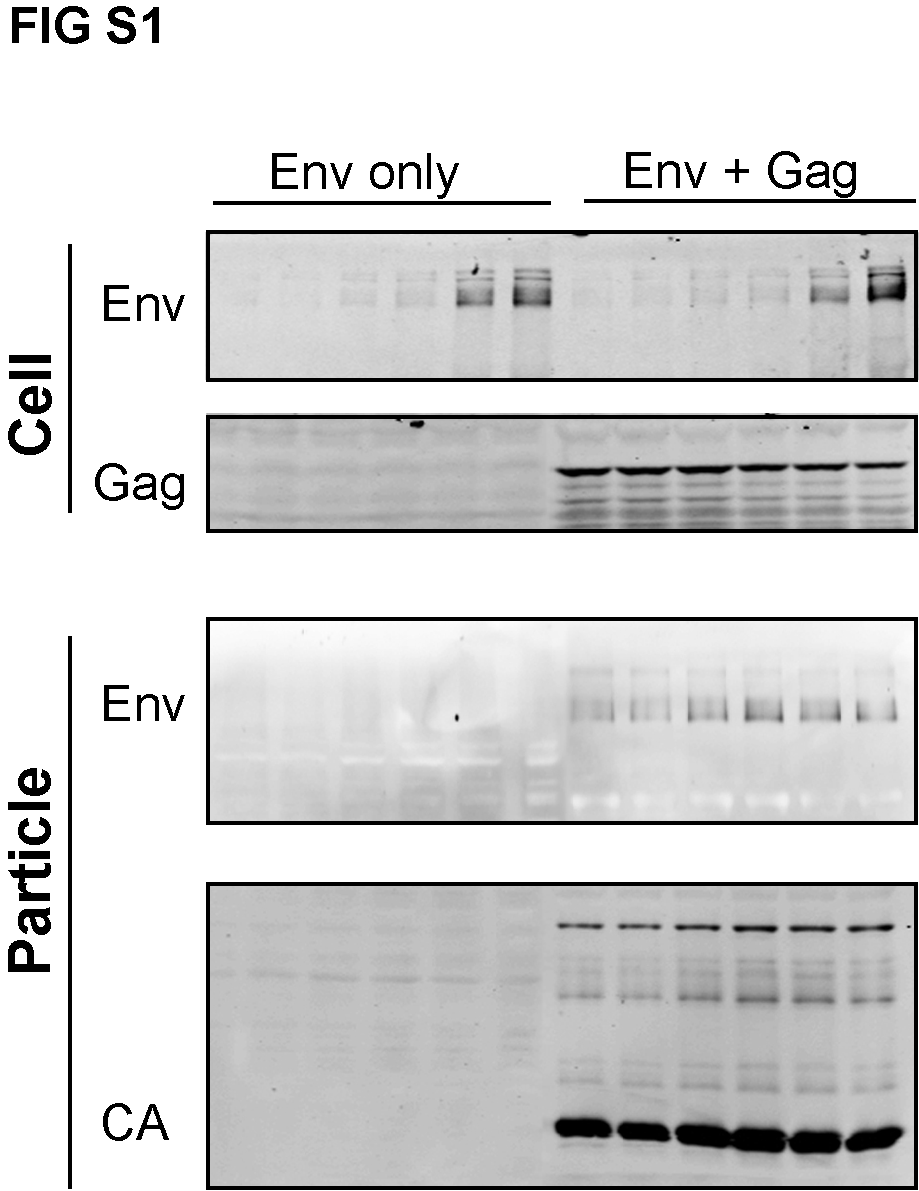

Supplement: Figure S1 — Env vs. Env + Gag pelleting. In order to confirm that overexpression of Env was not leading to pelletable Env in the absence of Gag, we performed a titration of Env expression in HeLa cells without (left) or with (right) a constant amount of Gag expression. Supernatant materials were pelleted through 20% sucrose cushions and analyzed by Western blotting for the proteins shown. (TIF) [file ppat.1003278.s001.tif]

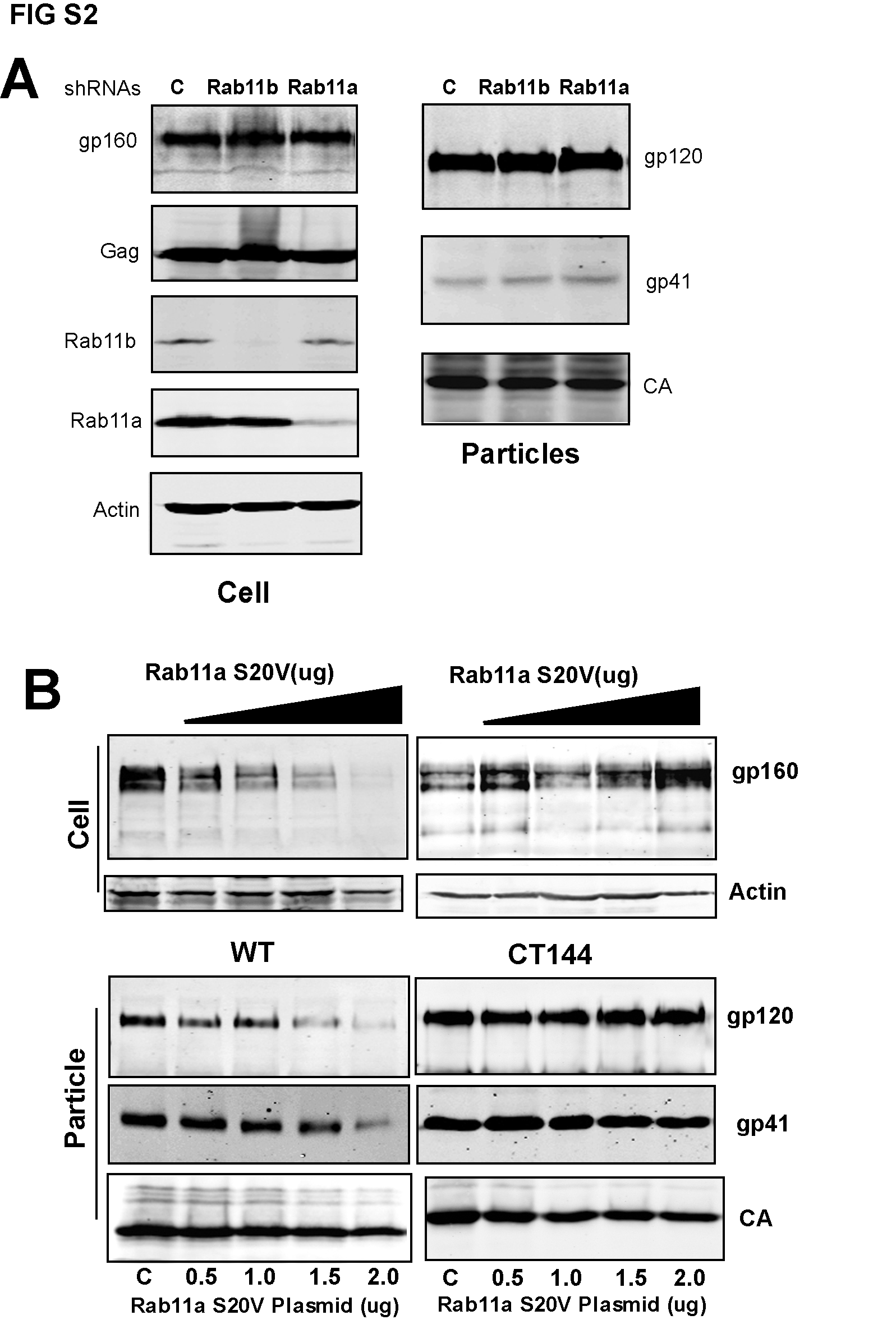

Supplement: Figure S2 — Recycling factor involvement in Env incorporation. A) Rab11a or Rab11b depletion does not alter Env incorporation. shRNA-mediated depletion of Rab11b (center lanes) or Rab11a (right lanes) was performed in HeLa cells, followed by expression of NL4-3 provirus. Cell lysates (left lanes) and pelleted viral particles (right) were analyzed by Western blotting for the indicated proteins. B) Overexpression of Rab11aS20V depletes particle-associated Env and leads to degradation of cellular Env. An expression plasmid for Rab11a S20V was titrated in HeLa cells co-expressing NL4-3 (left) or NL CT144 (right). Cellular Env levels and particle-associated Env were evaluated by Western blot. Full-length Env was depleted by S20V, while truncated Env was unaffected. (TIF) [file ppat.1003278.s002.tif]

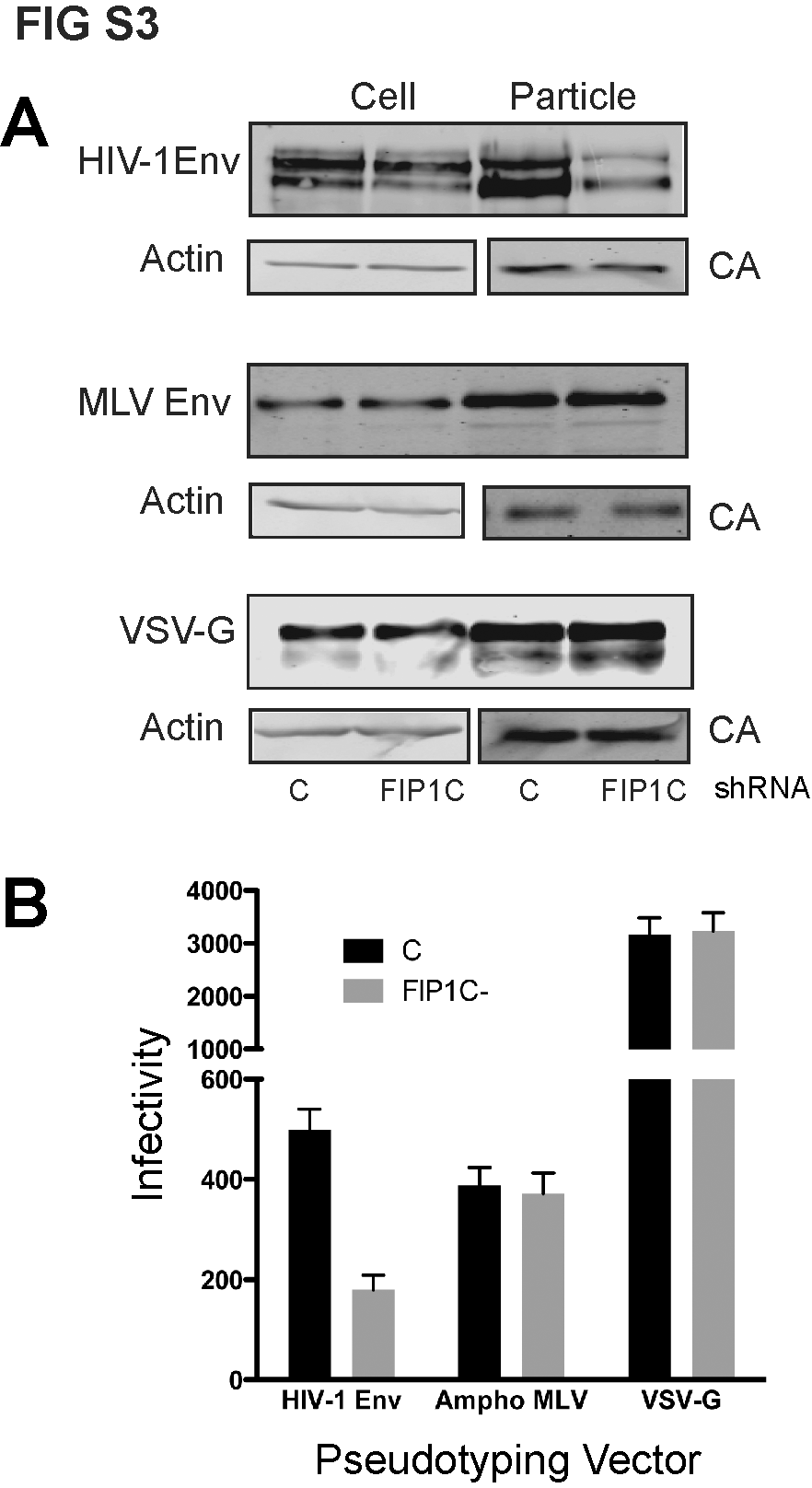

Supplement: Figure S3 — Effect of Rab11-FIP1C/RCP depletion on HIV particle pseudotyping with amphotropic MLV and VSV-G. (A) The effect of FIP1C/RCP depletion on amphotropic MLV Env (middle panel) and VSV-G protein (lower panel) incorporation onto HIV-1 particles was examined and compared with effects on gp120 particle incorporation (top). (B) Infectivity of pseudotyped viruses from (A) was measured using TZM-bl reporter cells; units are infected cells/ng p24 input. (TIF) [file ppat.1003278.s003.tif]

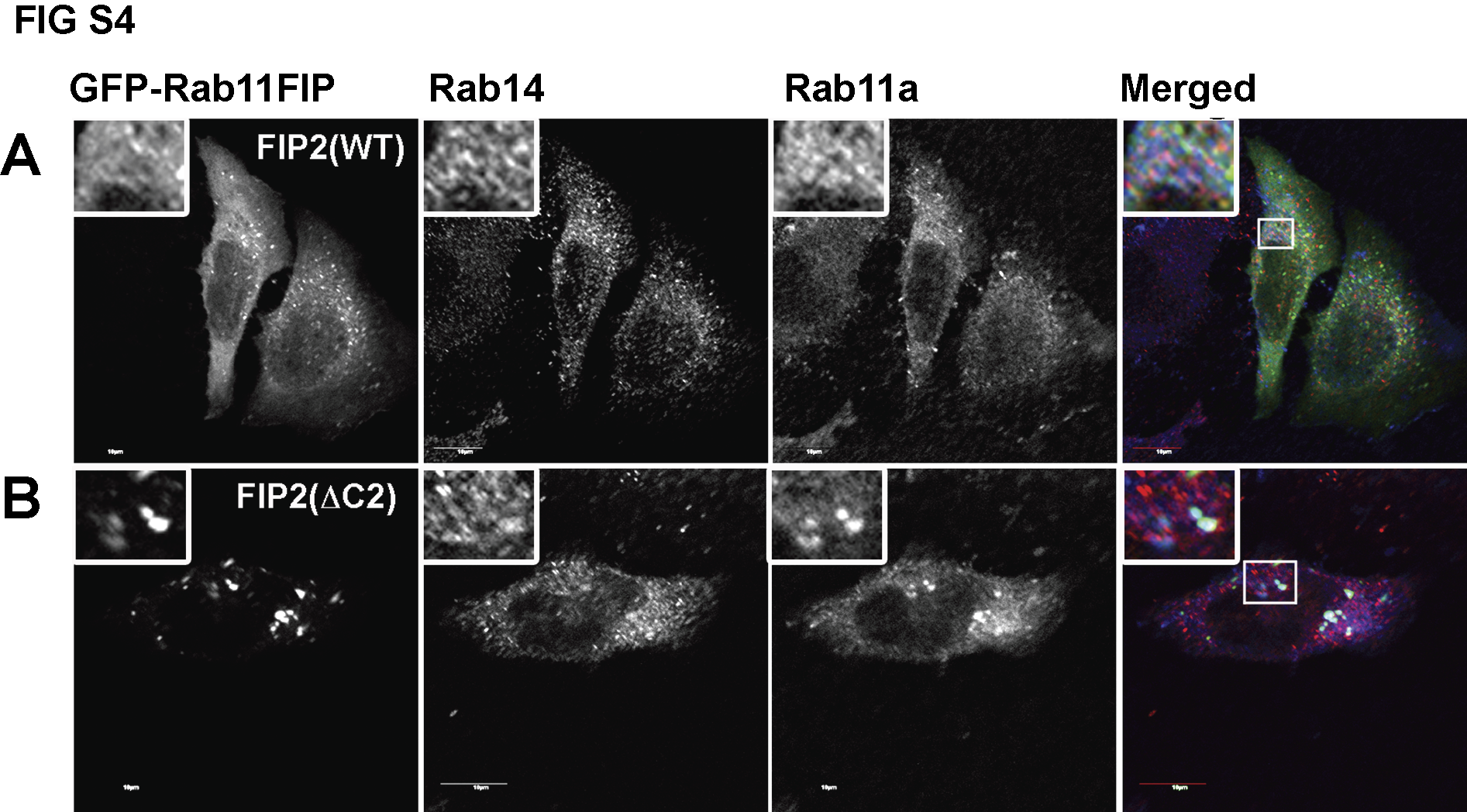

Supplement: Figure S4 — Rab14 does not associate with Rab11-FIP2. HeLa cells were transfected with either (A) wild type GFP-Rab11-FIP2 or (B) GFP-FIP2 (129–512), designated FIP2(ΔC2). Cells were fixed and then immunostained for endogenous Rab14 and endogenous Rab11a. Inserts show higher magnification regions. Note that FIP2(ΔC2) strongly concentrated Rab11a in association with the EGFP-chimera, but had no effect on Rab14 distribution. (TIF) [file ppat.1003278.s004.tif]

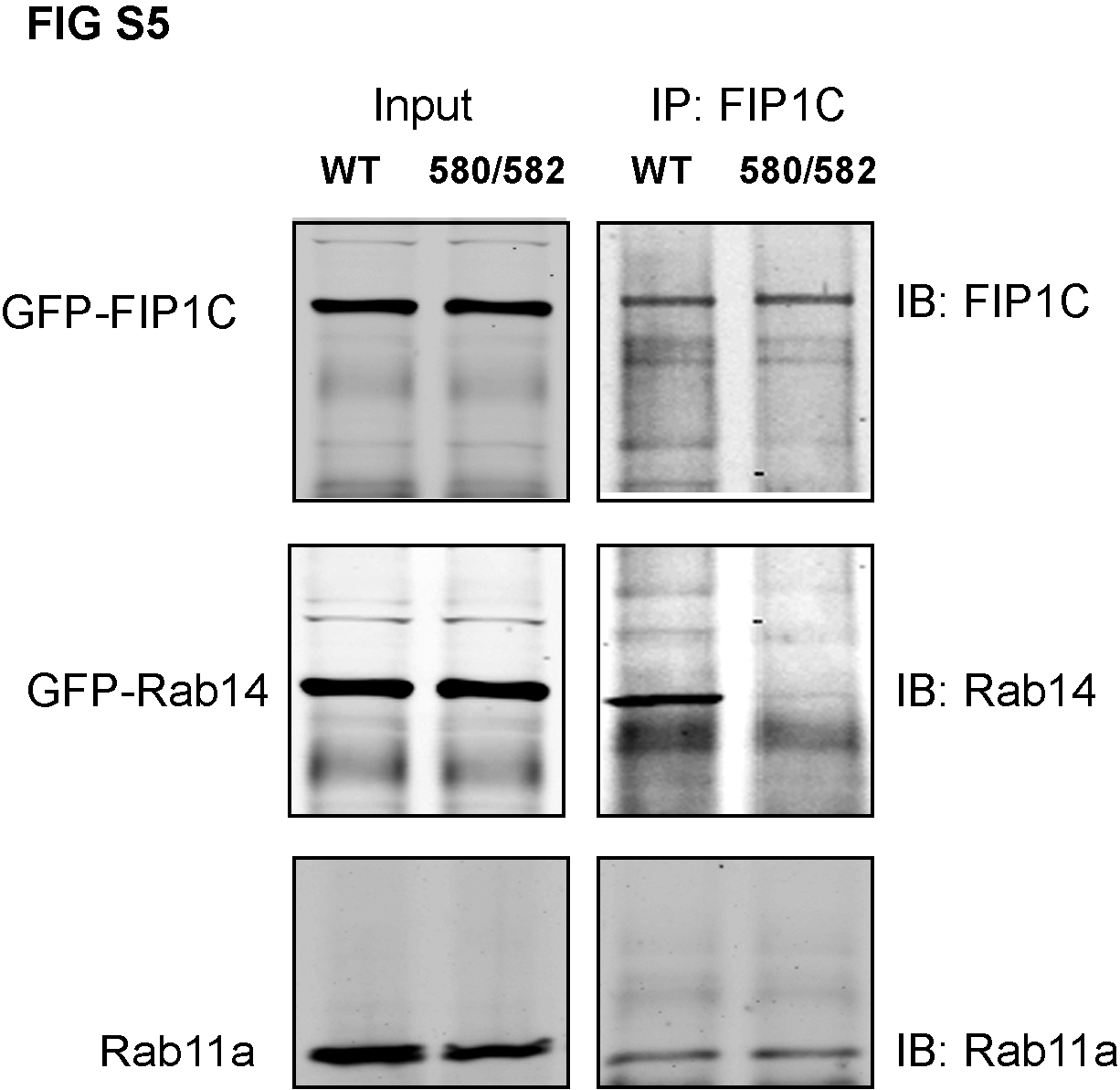

Supplement: Figure S5 — Co-immunoprecipitation of FIP1C with Rab14. HeLa Cells were transfected with EGFP-FIP1C WT or EGFP-FIP1C (S580N/S582L) and EGFP-Rab14. Input protein content is shown on left. IP was performed using FIP1C-specific antisera, followed by immunoblotting for the proteins indicated on the right. Endogenous Rab11a is shown. (TIF) [file ppat.1003278.s005.tif]

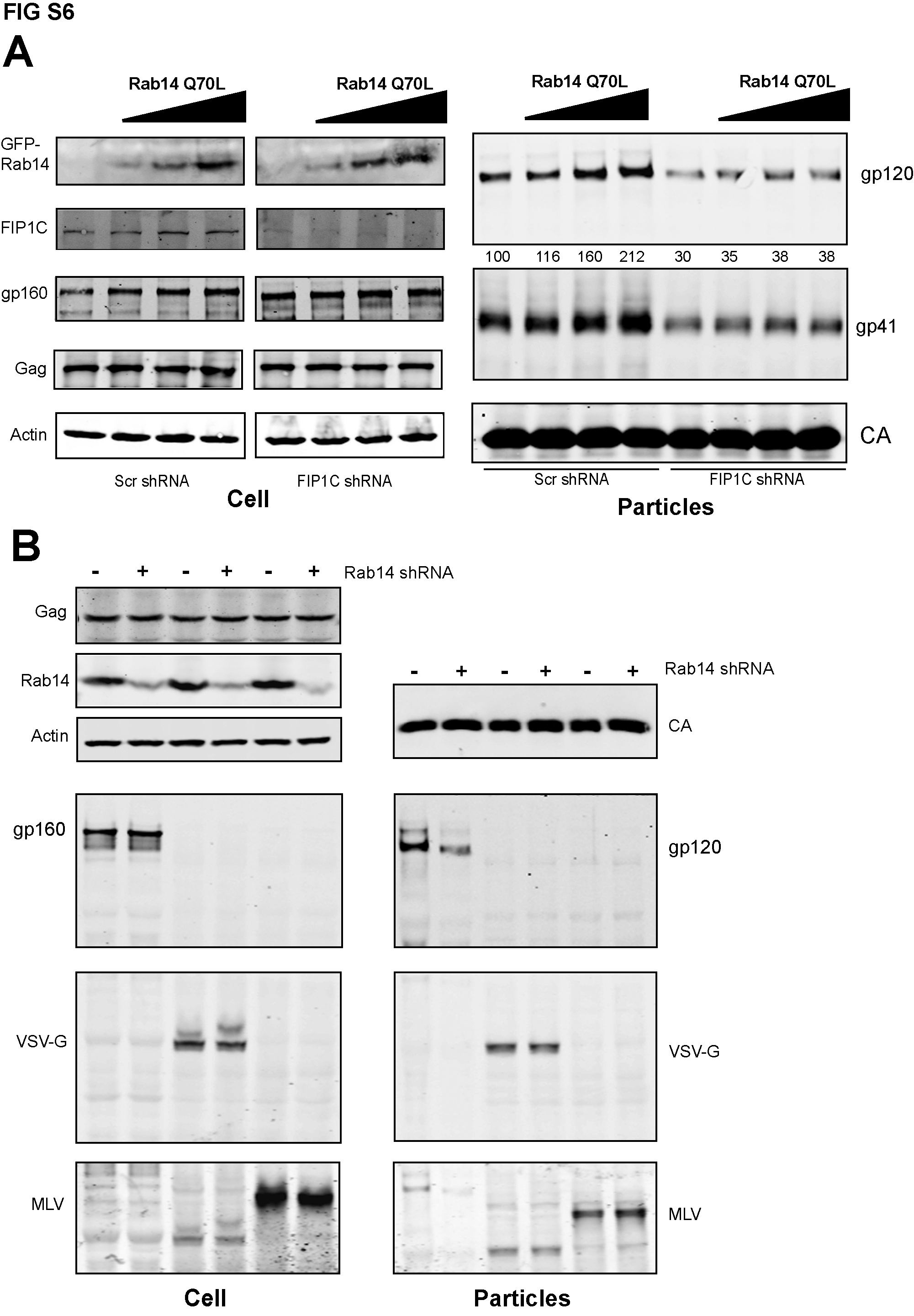

Supplement: Figure S6 — Rab14 enhancement of HIV-1 Env incorporation requires FIP1C and Rab14 depletion does not alter incorporation of MLV or VSV-G Env. A) Titration of Rab14Q70L in HeLa cells with normal FIP1C levels (Scr shRNA lanes) or in cells depleted of FIP1C (FIP1C shRNA lanes). Note that gp120 and gp160 blots were probed with goat anti-gp120/gp160 antisera, while gp41 was probed with murine monoclonal anti-gp41 antibody. B) Hela cells were transfected with NL4-3 or with NL4-3deltaEnv and expression constructs pHCMV-G (for VSV-G) or pCL-Ampho (MLV Env). Depletion of Rab14 was performed using shRNA in the indicated lanes. Cellular and particle Env content was assessed by immunoblotting with specific antisera for HIV gp120/160, VSV-G, or amphotropic MLV Env. (TIF) [file ppat.1003278.s006.tif]
