# Supplementary material for: Rab11-FIP1C and Rab14 Direct Plasma Membrane Sorting and Particle Incorporation of the HIV-1 Envelope Glycoprotein Complex
Source: PLoS Pathog. 2013 Apr 4;9(4):e1003278. doi: 10.1371/journal.ppat.1003278 (PMC3616983; doi:10.1371/journal.ppat.1003278)
Supplement: Text S1 — 1) Table S1: Split-ubiquitin yeast two-hybrid assay of Rab11-FIP protein interaction with Rab proteins. Split ubiquitin assays were performed between CCW vectors with Rab11-FIP proteins and DSL vectors with Rab proteins. Results were consistent over three separate experiments. 2) Supplemental Experimental Procedures. (DOC) [file ppat.1003278.s007.doc]

**SUPPORTING INFORMATION: TEXT S1**

Contents:
1) Supporting Table S1

3) Supplemental Experimental Procedures

**Supporting Table S1**

| **CCW-vector** | **DSL-Rab11a** | **DSL-Rab14** | **DSL-Rab8a** |
| --- | --- | --- | --- |
| Rab11-FIP1B | + | + | - |
| Rab11-FIP1C | + | + | - |
| Rab11-FIP2 | + | - | - |

**Supplemental Experimental Procedures**

**shRNA lentiviral-mediated depletion methods**. FIP1C shRNA-expressing pseudovirus was generated by cotransfecting 293T cells with the indicated shRNA construct (Sigma) and pHCMV-G. The shRNA was delivered into target cells following the manufacturer’s recommendations, using the pLKO.1-puro backbone vector. For infections, HeLa and H9 T cells were incubated with VSV-G-pseudotyped viral stocks or shRNA lentiviral stock at MOI 2 for 4 hours. Cells were then washed with PBS and incubated for 2 or 4 days for Hela and H9 respectively before harvesting for analysis. In some of the experiments, siRNAs were used to transiently knock down FIP1C/RCP in HeLa cells. Transfection experiments were performed in 293T and HeLa cells, which both were seeded in 10 cm^2^ dishes at a concentration of 2.0×10^6^ cells/dish and transfected the following day using either PEI (Sigma) at 4ug/ml final concentration or Lipofectamine 2000 (Invitrogen) for immunofluorescence studies.

**shRNA constructs, siRNA sequences and real-time PCR reagents.** ShRNA constructs targeting Rab11a-FIPs were purchased from Sigma : FIP1C/RCP shRNA ( TRCN0000145281, Sequence: CCGGGAAATCCAAACCAGGAAAGAACTCGAGTTCTTTCCTGGTTTGGATTTCTTTTTTG ); FIP2 shRNA (TRCN0000144035,Sequence: CCGGCCGCAAGTATGTTTGACTTATCTCGAGATAAGTCAAACATACTTGCGGTTTTTTG); FIP3 shRNA (TRCN0000056474,Sequence: CCGGCATCCAGTTTGCTACGGTCTACTCGAGTAGACCGTAGCAAACTGGATGTTTTTG). FIP4 shRNA (TRCN0000056504, Sequence: CCGGGCACGTGTACAACAGCGAATTCTCGAGAATTCGCTGTTGTACACGTGCTTTTTG); FIP5 shRNA (TRCN0000007153, Sequence: CCGGGTTTGGCTTGAGAGAAGCCAACTCGAGTTGGCTTCTCTCAAGCCAAACTTTTT). The shRNA for Rab11a, Rab11b, and Rab14 were also attained from Sigma: Rab11a (TRCN0000073020, Sequence: CCGGCCTGTCTCGATTTACTCGAAACTCGAGTTTCGAGTAAATCGAGACAGGTTTTTG); Rab11b (TRCN0000029184, sequence: CCGGGCCTTGGATTCCACTAACGTACTCGAGTACGTTAGTGGAATCCAAGGCTTTTT); Rab14 (TRCN0000293272, sequence: CCGGCACTAGAAGAAGTACATATAACTCGAGTTATATGTACTTCTTCTAGTGTTTTTG).

For transient knockdown experiments, siRNAs were custom-designed and purchased from Dharmacon. Sequences were: siRNA1- ggccugcugaagaccuuguguuu; siRNA2- agccccucggacccugcauuuuu. To quantify the mRNA level in HeLa cells and H9 T cells, real-time PCR primers targeting Rab11a-FIPs were designed as following: FIP1 forward- GCCTACGCGCAGCTGACCCACGAT and reverse- CACCTGGAACTCCTTCTTGCTTAT; FIP2 forward- AGATAGCAGAAGATCTGATAAAC and reverse- CACTAAATGCATTAGGTGATTTCA; FIP3 forward- CCTGCAGCTGGTGCACAGAGCAA and reverse- CTCTCAGCTCCTGCTCCTTCAG; FIP4 forward- TCTGGCCTAGGCGAGTTCAATG and reverse-CCGCAGCTTATAATTCTCCTGCTT; FIP5 forward-CAAGAGTAGCTGGTTTGGCTTGA and reverse-GGACTGGGTTTCTGAGTCGGGTC. To generate the shRNA-resistant GFP-FIP1C construct in the pEGFP-N3 vector (Clontech), we used overlap extension PCR methods and introduced a SmaI site into the cDNA for easy identification. The primers used were: N3-EcoRI-FIP1C forward: ACGAATTCTATGTCCCTAATGGTCTCGGCTGG; N3-KpnI-FIP1C reverse: ACGGTACCCATCTTTCCTGCTTTTTTGCCAAC; SmaI-281F: GTTGAAGTCAAAGCCCGGGAAAAAAGACAAGGAGCGAGGAGAAAT; SmaI-281R: TGTCTTTTTTCCCGGGCTTTGACTTCAACTTATACCACTGCGTCTTCCTGC. Similarly, overlap extension PCR mutagenesis was employed to generate the S580N /S582L FIP1C mutant. Two primers used here are 580/582 forward- GTCATGATGAAGAAATACAACCCCTTGGACCCTGCATTTGC and 580/582 reverse-GCAAATGCAGGGTCCAAGGGGTTGTATTTCTTCATCATGAC. The resulting amplicon was cloned into pEGFP-N3 vector using EcoR1 and Kpn1 restriction sites, creating a C-terminal GFP tag. To further study the FIP1C-Rab14 interaction and confirm the FIP1C repletion experiment, we also generated two shRNA-resistant FIP1C constructs with an N-terminal GFP tag. These were created at because the C-terminal GFP tag appeared to partially mislocalize the FIP1C constructs. C1-GFP-shRNAFIP1C WT and C1-GFP-shRNAFIP1C 580N/582L were generated by PCR mutagenesis using primer pairs: C1-FIP1C-EcoRI forward: ACGAATTCTATGTCCCTAATGGTCTCGGCTGG and C1-FIP1C-BamHI reverse: ACGGATCCCGGGCCCGCGGTACCCATCTTTCCTGCTTTTTTGCCAAC. Amplicons were purified, digested with EcoR1 and BamH1, and cloned into the EcoR1 and BamH1 sites of the pEGFP-C1 vector (Clontech). We also constructed the FIP1C mutants FIP1C*(D622N) and FIP1C*(I621E) by overlap extension PCR using the shRNA-resistant FIP1C as template, the overlapping mutagenesis primers used were: FIP1C622N-foward: TCCGCGAGCTGGAAGACTACGAAAACAACCTGCTTGTCAGGGTCATG; FIP1C622N-reverse: CATGACCCTGACAAGCAGGTTGTTTTCGTAGTCTTCCAGCTCGCGGA;

FIP1CI621E-forward: tccgcgagctggaagactacgaagacaacctgcttgtcagggtcatg; and FIP1CI621E-reverse: catgaccctgacaagcaggttgtcttcgtagtcttccagctcgcgga. Outer primer pairs were C1-FIP1C-EcoR1 forward and C1-FIP1C-BamH1 reverse and cloning was into pEGFP-C1 as before.

**Immunofluorescence staining protocol for Rab14 and FIP1C interaction analysis.** HeLa cells were plated on glass coverslips at a density of 60,000 cells per well of a Costar 12 well dish. Cells were grown 24 hours at 37°C with 5% CO_2_ then transfected using Effectene (Qiagen) transfection reagent using 150 ng of DNA of each construct. Samples were then incubated for 15-18 hours at 37°C with 5% CO_2_ prior to fixing in 4% Paraformaldehyde (PFA). After a brief 1X PBS wash, the cells were fixed for 15 minutes at room temperature, then washed 3 times in 1X PBS. Cells were then permeabilized in 1XPBS and 0.1% Tween-20 (BioRad) for 10 minutes at room temperature. Cells were then blocked for 30-60 minutes in 1XPBS, 10% normal donkey serum (NDS; Jackson Immuno Research) and 5% bovine serum albumin (BSA; Sigma Aldrich). After blocking, the cells were incubated in 1XPBS, 0.1%NDS and 0.5% BSA (incubation buffer) with the appropriate dilution of primary antibodies for at least 60 minutes at room temperature in humidity chamber. Primary antibody used for detection of endogenous Rab11 was 8H10 and for Rab14 was AVARP13107_P050 from Aviva Systems Biology. After primary antibody incubation, the cells were washed 3 times for 10 minutes with incubation buffer. The appropriate, Cy3 or Cy5 (Jackson Immuno Research) secondary antibodies were then diluted to 1:200 in incubation buffer and incubated at room temperature for 60 minutes in the humidity chamber. Cells were again washed 3 times for 10 minutes in incubation buffer and briefly rinsed with distilled water before being mounted in Prolong with DAPI (Molecular Probes) on glass slides. Cells were imaged using the Olympus Fluoview confocal microscope. Images were taken with 60X or 100X magnification objectives.

**Surface biotinylation reaction for HIV Envelope.** Transfected cells were washed in PBS and incubated for 30 minutes at room temperature with 10mM NHS-SS-Biotin (Thermal Scientific). The reaction was then quenched by adding complete media for 5 minutes. Labeled cells were washed and lysed. Lysates were clarified and then incubated with streptavidin beads. Pelleted beads were washed and bound proteins eluted in loading buffer for SDS-PAGE. Blotting was performed with goat anti-gp120/gp160 antisera as before.

**Split-ubiquitin Yeast Two-hybrid**. Split ubiquitin yeast two-hybrid analyses were performed according to the methods of Iyer et al. . Bait and prey constructs were transformed into the yeast strain NMY32, plated on −Trp−Leu selection plates, and incubated at 30 °C for 3–4 days. Approximately five medium-size colonies were selected and resuspended in water. After all samples were diluted to equivalent densities, serial dilutions were spotted on selection plates −Trp/−Leu, −Trp/−Leu/−His, and −Trp/−Leu/−His/+10 mM 3-amino-1,2,4 triazole. After 3–4 days of growth at 30 °C, the presence of protein interactions was determined using the most stringent selection. The results shown are representative of at least three trials.

**Supporting Information References**

1. Goldenring JR, Smith J, Vaughan HD, Cameron P, Hawkins W, et al. (1996) Rab11 is an apically located small GTP-binding protein in epithelial tissues. The American journal of physiology 270: G515-525.

2. Iyer K, Burkle L, Auerbach D, Thaminy S, Dinkel M, et al. (2005) Utilizing the split-ubiquitin membrane yeast two-hybrid system to identify protein-protein interactions of integral membrane proteins. Science's STKE : signal transduction knowledge environment 2005: pl3.
